# Chemoproteomic Identification of AKT2 as a Paclitaxel‐Binding Protein via C─C Bond‐Linked Probe PTX‐4 in Paclitaxel‐Resistant Breast Cancer

**DOI:** 10.1002/advs.202520089

**Published:** 2026-07-30

**Authors:** Kai Wang, Yuqing Yuan, Wei Shen, Xiuxiu Yang, Xiaokang Wu, Haihua Zhou, Yi Hu, Qing Zhu

**Affiliations:** ^1^ State Key Laboratory of Green Chemical Synthesis and Conversion Hangzhou China; ^2^ Department of Surgery Affiliated Jinhua Hospital Zhejiang University School of Medicine Jinhua China; ^3^ State Key Laboratory of Complex Severe and Rare Diseases Biomedical Engineering Facility of National Infrastructures For Translational Medicine Institute of Clinical Medicine Peking Union Medical College Hospital Chinese Academy of Medical Sciences and Peking Union Medical College Beijing China

**Keywords:** breast cancer, chemical proteomics, paclitaxel, photoaffinity probes, PTX resistance

## Abstract

Breast cancer remains one of the most prevalent malignancies among women, and taxane‐based chemotherapies such as paclitaxel and docetaxel are central to standard treatment regimens. However, drug resistance in breast cancer limits therapeutic efficacy and contributes to recurrence and metastasis. Identifying resistance‐associated molecular targets is therefore critical for advancing treatment strategies. To investigate paclitaxel resistance, we designed and synthesized four paclitaxel‐derived probes. Among these, PTX‐4, constructed via a stable C─C bond linkage, exhibited superior efficiency. Using a chemoproteomic approach, we systematically profiled paclitaxel‐binding proteins in parental and resistant breast cancer cells. This strategy successfully identified AKT2, an unrecognized paclitaxel‐interacting protein in paclitaxel‐resistant breast cancer. Functional validation demonstrated that AKT2 is a direct target of paclitaxel in paclitaxel‐resistant cells. Knockdown and pharmacological inhibition of AKT2 restored the sensitivity of paclitaxel‐resistant cells to paclitaxel. These findings establish AKT2 as a key mediator of paclitaxel resistance in breast cancer. Targeting AKT2 may offer a promising therapeutic strategy to overcome resistance and improve the clinical efficacy of taxane‐based chemotherapy. This study highlights the advantage of C─C bond‐linked, pharmacologically active probes for chemoproteomic profiling of drug targets.

## Graphical abstract

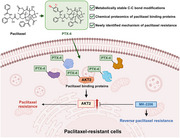

## 1 Introduction

Breast cancer is one of the most common and life‐threatening malignancies affecting women globally, with incidence rates steadily rising [1]. Current clinical management includes surgery, radiotherapy, chemotherapy, targeted therapy, immunotherapy, and combination regimens [2]. Among chemotherapeutic agents, paclitaxel (PTX) remains a first‐line treatment for both early‐stage and metastatic breast cancer, demonstrating substantial therapeutic efficacy [3]. Nevertheless, overall survival remains suboptimal, largely due to the frequent development of chemoresistance [4]. Notably, drug resistance leads to treatment failure in over 90% of patients with advanced disease [5]. Drug resistance involves diverse mechanisms, including enhanced drug efflux, augmented DNA repair, reduced apoptosis, and altered drug metabolism [6]. Given the limited pipeline of novel chemotherapeutics, research emphasizes overcoming resistance through combination therapies. PTX‐based combinations have exhibited promising potential [7]. For example, Wan et al. developed PTX/cisplatin co‐loaded polymeric micelles that demonstrated superior efficacy against drug‐resistant breast cancer compared with monotherapies [8]. Similarly, Xiong et al. reported that a nanosystem co‐delivering PTX and curcumin significantly suppressed tumor growth and improved survival in murine models compared to free drugs [9]. Collectively, these findings highlight combination regimens as a rational and effective strategy to circumvent breast cancer drug resistance.

At the mechanistic level, PTX primarily exerts cytotoxicity by binding to tubulin, stabilizing microtubules, inducing mitotic arrest, and ultimately triggering apoptosis [10]. Importantly, beyond this classical mechanism, accumulating evidence indicates that PTX can also modulate the expression and activity of drug resistance‐related proteins, including P‐glycoprotein (ABCB1), Bcl‐2 family members, and survivin [11]. Numerous studies have investigated these indirect resistance factors. However, the cellular networks involved in paclitaxel resistance are highly complex, making it difficult to identify the most critical targets from indirect factors alone. In comparison, proteins that directly bind to PTX are often more important because they act at the very beginning of drug action. In resistant cells, these direct binding proteins might act as a sponge to capture the drug and reduce its interaction with tubulin. They can also directly trigger downstream survival pathways under drug pressure. Therefore, identifying these non‐tubulin direct targets is crucial, as it allows us to discover the primary driving factors of resistance that can be directly targeted. Thus, elucidating the direct molecular targets of PTX is essential for guiding the rational design of next‐generation therapies that more effectively overcome resistance.

Chemical proteomics offers such a platform, providing an unbiased means to map drug–protein interactions in complex cellular contexts [12]. Among its core methodologies, affinity‐based protein profiling (AfBPP) integrates affinity‐based probes (AfBPs) with high‐resolution mass spectrometry, enabling comprehensive identification of protein targets for bioactive molecules [13]. AfBPP has been successfully applied to the anticancer drug doxorubicin [14], the antimalarial artemisinin [15], and the natural product celastrol [16], revealing previously unrecognized mechanisms of action. However, few AfBPP studies have been reported for PTX to date. This limitation is largely attributed to conventional probe designs that rely on ester‐bonded linkers. While synthetically accessible, these linkages are highly susceptible to cleavage by endogenous esterases. To overcome this, the construction of probes via robust C‐C bonds is essential, as such metabolic stability ensures the integrity of the probe‐target complex and significantly enhances both binding specificity and experimental reliability in complex biological environments [17].

In this study, we report the synthesis of the first C─C bond‐linked PTX activity probes, designed to overcome instability issues and achieve high‐specificity target identification. By combining photoaffinity labeling with proteomic analysis, we systematically identified the direct protein targets of PTX in breast cancer cells. Furthermore, using a PTX‐resistant cell line model, we identified AKT2, a key kinase in the PI3K/AKT pathway, as a critical mediator of resistance. These findings provide new molecular evidence for understanding PTX resistance and suggest AKT2 as a promising therapeutic target. Collectively, this work establishes a foundation for rational strategies aimed at enhancing PTX efficacy in resistant breast cancer.

## 2 Results

### 2.1 Rational Design of PTX‐Derived Probes

**FIGURE 1:**
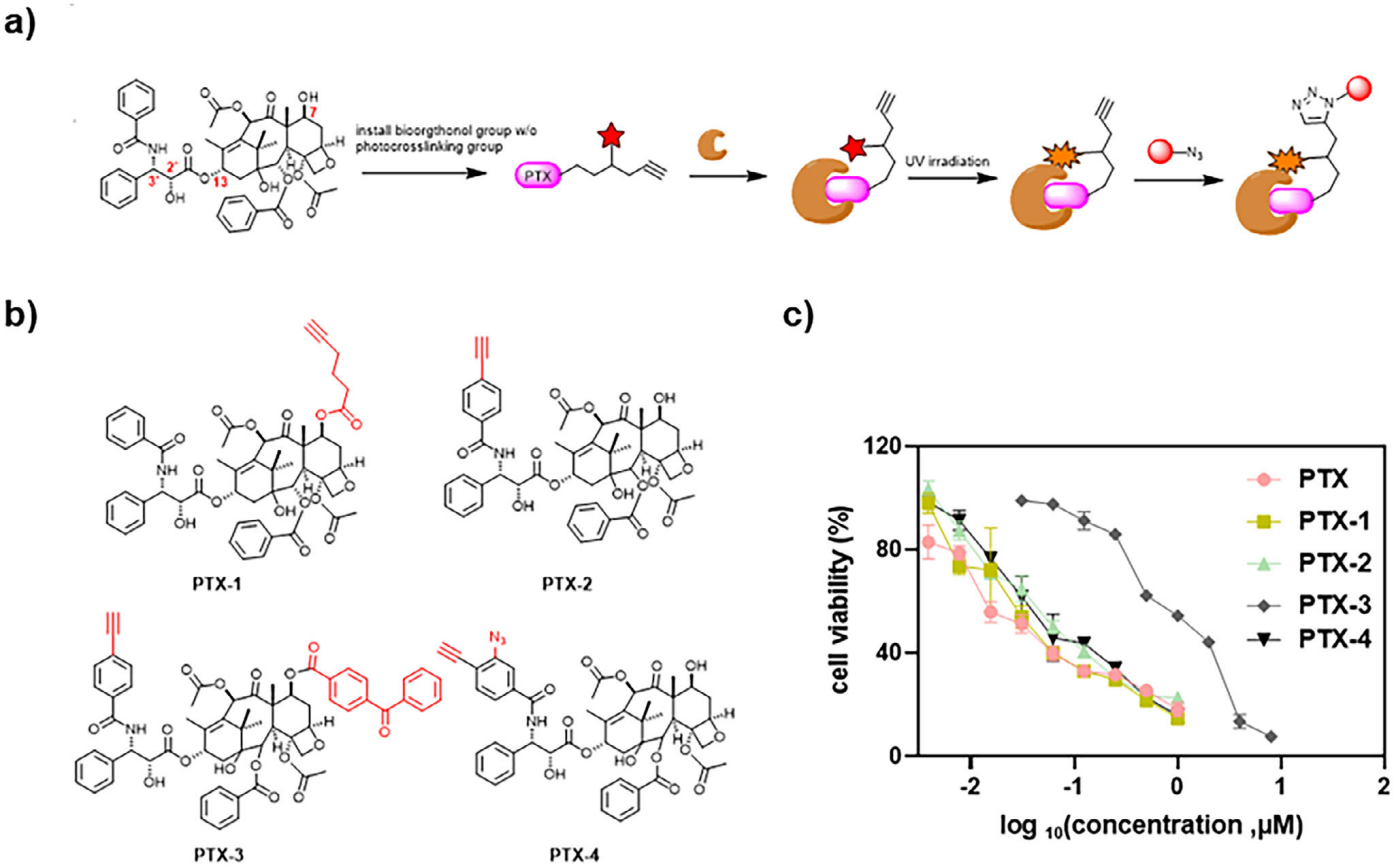
(a) Chemoproteomic analysis of PTX target proteins. (b) Structure of the PTX affinity‐based probes. (c) Evaluation of the antitumor activity of PTX and the probes in MCF‐7 cells.

The design of effective affinity‐based probes requires careful selection of modification sites that preserve the parent drug's pharmacological activity [18, 19]. For PTX, extensive structure–activity relationship (SAR) studies have demonstrated that the C13 phenylisoserine side chain is indispensable for microtubule‐stabilizing activity, while the C2′ hydroxyl group is essential for maintaining maximal cytotoxic potency. In contrast, modifications at the C7 position are generally well tolerated, making it an attractive locus for probe engineering (Figure 1a). Guided by these principles, we designed and synthesized four PTX‐derived probes (PTX‐1 to PTX‐4) incorporating bioorthogonal handles and photo‐crosslinking groups with distinct structural features (Figure 1b).

PTX‐1 was synthesized by esterification at the C7 position to introduce an alkyne group, providing a bioorthogonal handle for subsequent conjugation. To enhance the stability and specificity of target capture, we next designed PTX‐2, in which an alkyne moiety was introduced at the phenyl ring of the C13 side chain through a stable C‐C bond. To enable covalent capture of binding partners, PTX‐3 incorporated a benzophenone photo‐crosslinker at the C7 position, leveraging its ability to generate reactive intermediates under UV irradiation. Finally, PTX‐4 was designed to maximize the retention of PTX activity by combining an alkyne bioorthogonal group with a minimal azide photo‐crosslinker, both integrated via a stable C─C bond at the C3' phenyl ring. All four probes were synthesized as described in the Supporting Information, and their structures were confirmed by 1H and 13C NMR spectroscopy.

### 2.2 Evaluation of Antiproliferative Activity

To assess whether the modifications affected biological activity, the antiproliferative effects of PTX and the probes were evaluated in MCF‐7 human breast cancer cells (Figure 1c). Consistent with previous reports, PTX exhibited potent cytotoxicity with an IC_50_ value of 0.042 µM. Among the probes, PTX‐1 displayed nearly equivalent potency (IC_50_ = 0.051 µM), indicating that ester‐linked C7 modification was well tolerated. PTX‐2 and PTX‐4 showed slightly reduced activity (IC_50_ = 0.081 and 0.082 µM, respectively), yet remained in the submicromolar range, confirming their suitability for functional studies. By contrast, PTX‐3 exhibited markedly diminished activity (IC_50_ = 1.129 µM), suggesting that the steric bulk of the benzophenone group at C7 disrupts optimal drug–target interactions.

Together, these results demonstrate that three of the four designed probes (PTX‐1, PTX‐2, and PTX‐4) retained sufficient cytotoxic activity to serve as viable PTX surrogates, fulfilling the basic requirements for subsequent affinity‐based protein profiling and chemical proteomics. These probes provide complementary platforms for interrogating PTX–protein interactions with varying combinations of bioorthogonal handles and photo‐crosslinking chemistries.

### 2.3 Establishment and Characterization of PTX‐Resistant MCF‐7/PTX Cells

**FIGURE 2:**
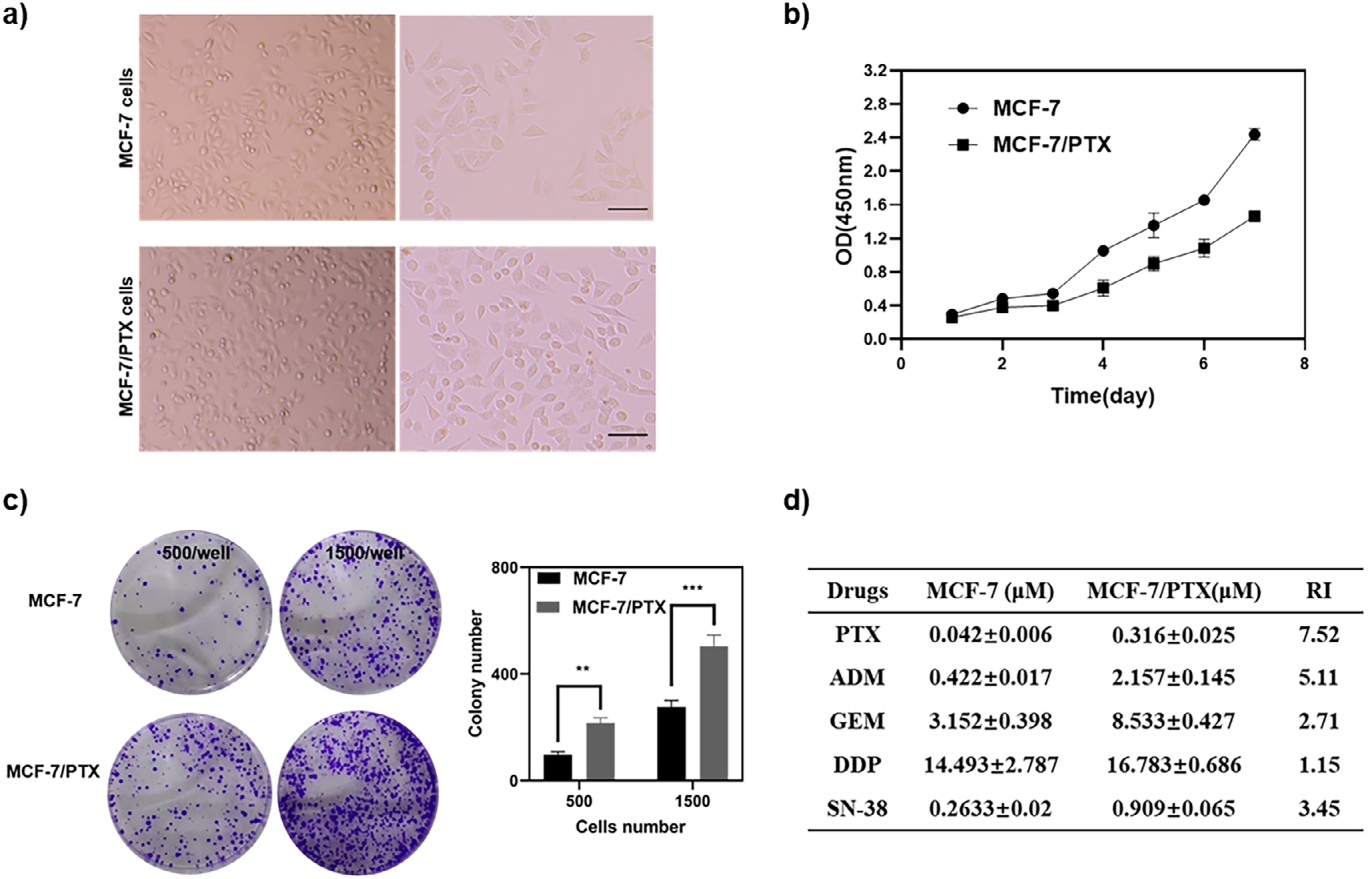
(a) Morphological differences between MCF‐7 and MCF‐7/PTX cells observed under optical microscopy at low magnification (left) and high magnification (right). Scaled bar = 30 µm. (b) Growth curves of MCF‐7 and MCF‐7/PTX cells. (c) Colony formation assay of MCF‐7 and MCF‐7/PTX cells treated with PTX. (d) IC_50_ values and resistance indices of different chemotherapeutic agents in MCF‐7 and MCF‐7/PTX cells. Data are shown as mean ± SD, *n* = 3, ^**^ *p* < 0.05, ^***^ *p* < 0.001. *IC_50_ values were calculated using GraphPad Prism software with a four parameter logistic regression model*.

To investigate PTX resistance–associated proteins, we established a stable PTX‐resistant breast cancer cell line (MCF‐7/PTX) using a stepwise concentration gradient induction strategy. Parental MCF‐7 cells served as the starting model and were initially exposed to a low PTX concentration (0.02 µM). The drug concentration was gradually escalated over an 8‐month period of continuous exposure, ultimately generating a resistant subline capable of stable proliferation in the medium containing 0.5 µM PTX. Morphological comparison revealed striking differences between parental and resistant cells. While parental MCF‐7 cells displayed typical adherent growth with uniform, spindle‐ or polygonal‐shaped morphology arranged in an orderly fashion, resistant cells acquired distinct features during drug selection. Following initial massive cell death, a minority of cells survived and gradually adapted to sustained drug pressure. These resistant cells exhibited increased vacuolization, elongated morphology, and a tendency to form compact clusters (Figure 2a).

Growth curve analysis further quantified these phenotypic differences. The parental MCF‐7 line exhibited a doubling time of 33.58 ± 0.29 h, whereas MCF‐7/PTX cells demonstrated a significantly prolonged doubling time of 40.04 ± 2.33 h, indicating that the acquisition of PTX resistance was accompanied by a notable reduction in proliferative capacity (Figure 2b). These findings are consistent with previously described biological characteristics of PTX‐resistant cells established by long‐term drug exposure, which typically display slower growth kinetics [20].

To validate the resistant phenotype, we performed functional assays assessing clonogenic survival and drug sensitivity. Colony formation assays revealed that MCF‐7/PTX cells maintained robust proliferative capacity under PTX treatment, in sharp contrast to the growth inhibition observed in parental cells (Figure 2c). Drug sensitivity profiling confirmed a resistance index (RI) of 7.52 for PTX, indicative of strong acquired resistance. Moreover, cross‐resistance patterns were observed: MCF‐7/PTX cells displayed high resistance to adriamycin (ADM, RI = 5.11), moderate resistance to gemcitabine (GEM, RI = 2.71) and SN‐38 (RI = 3.45), while retaining sensitivity to cisplatin (Figure 2d). This PTX resistance phenotype aligns with characteristic profiles of chemotherapy‐resistant breast cancer cells reported in vitro [21].

To further corroborate the multidrug resistance (MDR) phenotype and evaluate the functional activity of drug efflux pumps in our established model, intracellular accumulation assays were performed using three distinct fluorescent substrates [22]. As visualized by confocal microscopy, MCF‐7/MDR cells exhibited significantly lower intracellular fluorescence compared to parental MCF‐7 cells when incubated with Rhodamine 123, Doxorubicin (DOX), and Calcein‐AM (Figure S1). This marked reduction in substrate retention confirms the enhanced efflux capacity of the resistant subline, a hallmark of classical MDR. Collectively, these functional data, along with the drug sensitivity profiles, rigorously validate the reliability of the MCF‐7/MDR model for subsequent chemoproteomic target identification.

Collectively, these findings confirm the successful establishment of a stable PTX‐resistant breast cancer model. The MCF‐7/PTX cell line not only reproduces the hallmarks of drug resistance but also exhibits cross‐resistance to structurally and mechanistically distinct agents, thereby providing a reliable in vitro system for subsequent chemoproteomics‐based investigations into the molecular mechanisms underlying PTX resistance.

### 2.4 Optimizing PTX‐Derived Probes for Target Identification in Breast Cancer Cells

**FIGURE 3:**
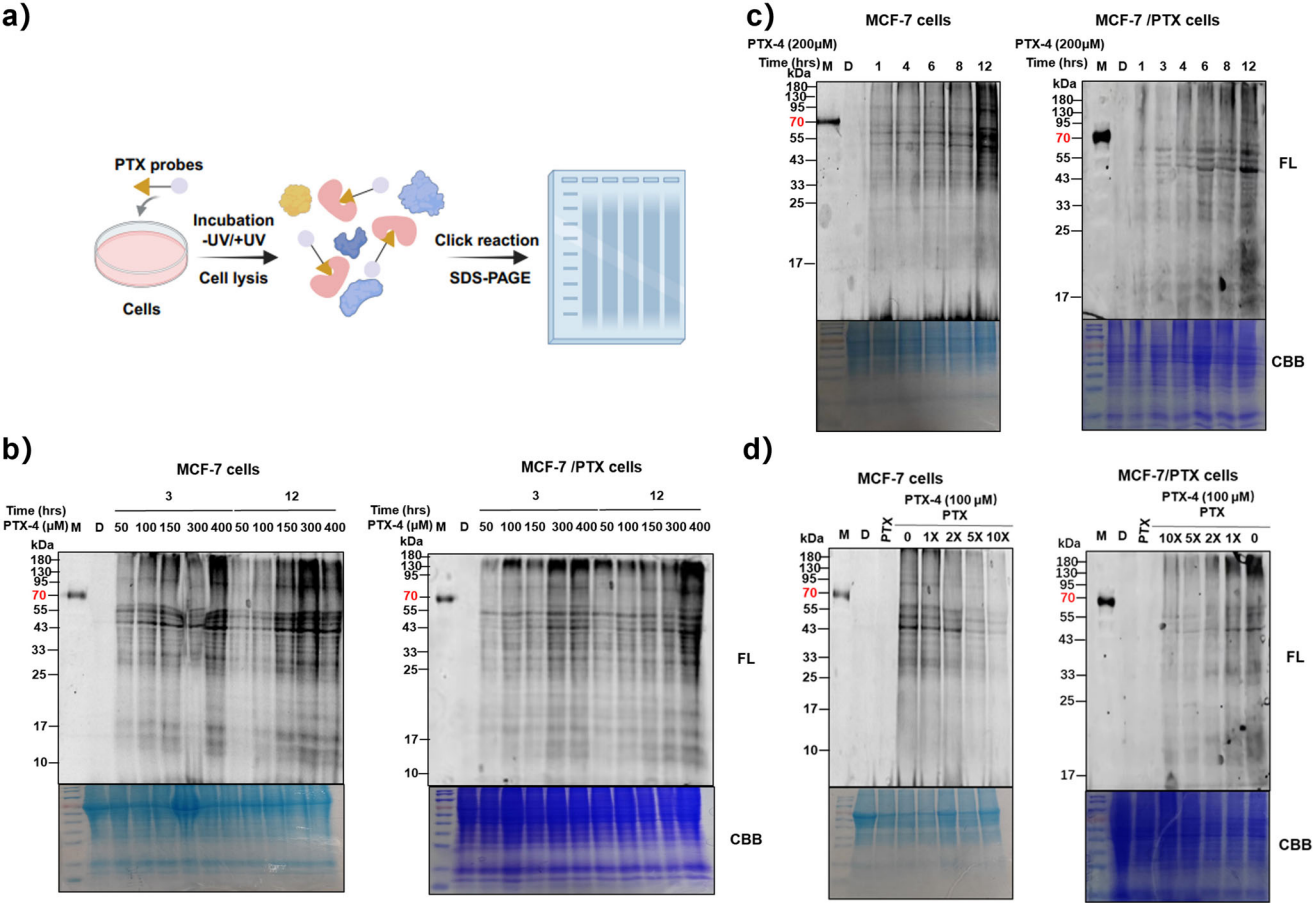
(a) Workflow of gel‐based analysis for PTX‐interacting proteins in live cells. (b) In‐gel fluorescence analysis of MCF‐7 and MCF‐7/PTX live cells labeled with PTX‐4 probe at different concentrations. (c) In‐gel fluorescence analysis of MCF‐7 and MCF‐7/PTX live cells labeled with PTX‐4 probe for different incubation time. (d) Competitive inhibition of PTX‐4 probe by PTX in live MCF‐7 and MCF‐7/PTX cells. FL, fluorescence imaging; CBB, Coomassie Brilliant Blue staining. M: Protein Marker; D: DMSO control.

To investigate the endogenous protein targets of PTX in their native cellular environment, we first assessed the labeling efficiency of four PTX‐derived active probes in living cells using a standard chemical proteomics workflow (Figure 3a). Both parental MCF‐7 and PTX‐resistant MCF‐7/PTX cells were incubated with the probes, followed by UV irradiation at 365 nm to crosslink PTX‐3 and PTX‐4 with their binding proteins. After cell lysis, probe‐labeled proteins were conjugated via click chemistry with rhodamine‐azide (Rh‐N_3_) for visualization, and fluorescence intensity was quantified through in‐gel scanning.

The initial results revealed that PTX‐2, PTX‐3, and PTX‐4 produced concentration‐dependent increases in labeling intensity in both cell lines, whereas PTX‐1 consistently exhibited low fluorescence signals with no significant changes (Figure 3b, Figure S2a,b and S3a). This poor labeling performance of PTX‐1 is likely attributable to its susceptibility to hydrolysis, arising from the ester bond at the C7 position linking PTX and the alkyne moiety in cellular environments [23]. In contrast, extending the incubation period to 12 h markedly enhanced the labeling signals of PTX‐2, PTX‐3, and PTX‐4, thereby confirming their superior intracellular protein‐labeling efficiency (Figure 3c, Figures S2d and S3b).

To further validate probe specificity, competition experiments were performed by pre‐treating cells with excess PTX prior to probe labeling. A dose‐dependent reduction in total fluorescence intensity was observed (Figure 3d, Figure S3c), indicating that the probes and PTX compete for the same protein binding sites. This finding provides direct evidence supporting the target specificity of the probes.

Taken together, these results highlight PTX‐4 as the most effective probe under the tested conditions. Not only did PTX‐4 exhibit strong labeling efficiency in both sensitive and resistant cell lines, but it also reliably captured PTX‐associated target proteins. Consequently, PTX‐4 was selected as the optimal probe for subsequent proteomic profiling of PTX‐resistant breast cancer cells.

### 2.5 Proteomic Profiling and Validation of PTX‐Interacting Proteins in Sensitive and Resistant Breast Cancer Cells

**FIGURE 4:**
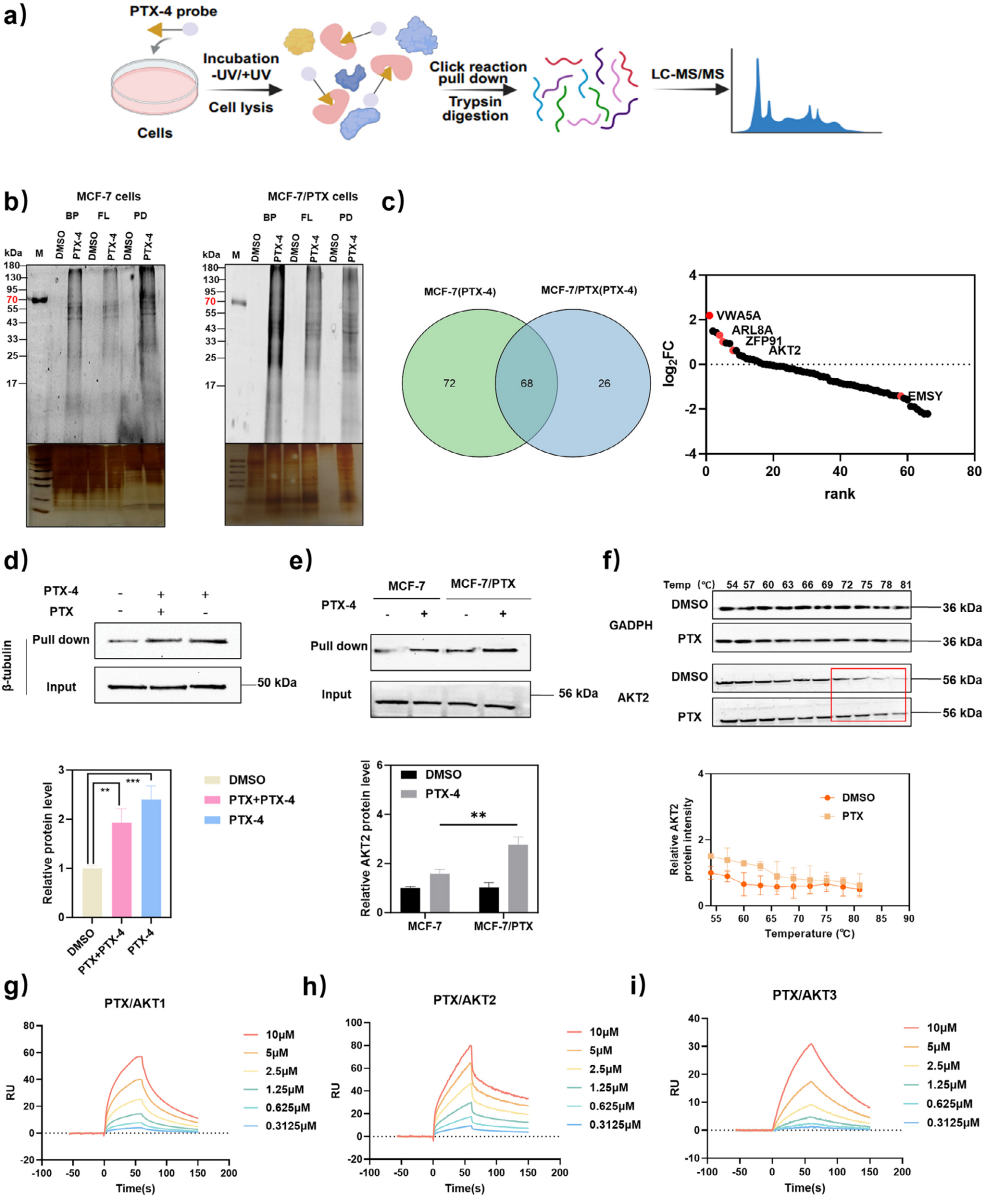
(a) Workflow of the mass spectrometry‐based proteomic analysis for identifying PTX‐interacting proteins in live cells. (b) Protein enrichment during the intracellular pull‐down assay using the probe. (c) Venn diagram and scatter plot analysis of PTX‐4 probe‐enriched drug resistance‐associated proteins. (d) Validation of PTX‐4 interaction with the known protein β‐tubulin via pull‐down/western blot, along with grayscale statistical analysis. (e) Immunoblot validation and grayscale statistical analysis of the differentially expressed protein AKT2 after pull‐down. (f) CETSA analysis and statistical quantification of intracellular binding between PTX and AKT2 proteins. mean ± SD, *n* = 3; ^**^ *p* < 0.01, ^***^ *p* < 0.001. (g–i) Surface plasmon resonance (SPR) sensorgrams characterizing the direct binding kinetics between PTX and recombinant human AKT1 (g), AKT2 (h), and AKT3 (i) proteins. Uncropped images of Figure 4d–f of the gels are provided in Figures S9–S11.

Having established the optimal probe for target profiling, we next employed mass spectrometry (MS) to identify potential protein targets captured by PTX‐4. The overall workflow for protein target identification is illustrated in Figure 4a. MCF‐7 and PTX‐resistant MCF‐7/PTX cells were treated with PTX‐4 or DMSO (control). Following cell lysis, probe‐labeled proteins were conjugated to biotin‐rhodamine‐azide (Bio‐Rh‐N_3_) via click chemistry and enriched using streptavidin affinity pull‐down. Silver staining confirmed distinct enrichment of protein bands (Figure 4b), and subsequent LC‐MS/MS analysis in biological triplicates revealed the protein identities.

For comprehensive target identification, LC‐MS/MS datasets were further analyzed using stringent thresholds (probe/control ratio > 4 and *p* < 0.05) to distinguish specific binders from background proteins. In total, 140 candidate proteins were identified in MCF‐7 cells and 94 in MCF‐7/PTX cells, with 68 proteins shared between both groups (Figures S4 and 4c). Comparative analysis of these 68 common proteins revealed differential expression patterns in resistant cells: 14 proteins were upregulated (FC > 1.5), whereas 23 were downregulated (FC < 0.5). Functional enrichment analysis of the captured proteins provided deeper insight into their biological roles (Figure S5). KEGG pathway annotation revealed involvement in FcγR‐mediated phagocytosis, proteoglycan signaling, autophagy, and related processes. Gene Ontology (GO) analysis further highlighted their predominant roles in protein transport, DNA‐templated transcription, ER‐to‐Golgi vesicle trafficking, and integrin‐mediated signaling regulation. Subcellular localization analysis indicated enrichment in the cytoplasm, nucleus, and extracellular exosomes, while molecular function (MF) analysis confirmed their association with protein binding, transcription cofactor activation, microtubule binding, and iron ion binding. Together, these findings suggest that PTX‐interacting proteins participate in diverse yet critical cellular pathways, including those relevant to drug resistance.

Among the identified proteins, several significantly upregulated candidates drew particular attention, including VWA5A and ZFP91 alongside AKT2. The ranking analysis highlighted these proteins as top enriched binders in the resistant cell line. Functional enrichment analysis of the entire captured protein set provided deeper insight into their biological roles, revealing involvement in diverse yet critical cellular pathways such as autophagy and proteoglycan signaling. Gene Ontology analysis further confirmed that these proteins play predominant roles in protein transport, ER‐to‐Golgi vesicle trafficking, and integrin‐mediated signaling regulation. Within this proteomic landscape, AKT2 stands out as a key component of the PI3K/AKT signaling pathway. It is well recognized for promoting resistance to multiple chemotherapeutic agents when overexpressed. Thus, the identification of AKT2, together with other differentially expressed proteins, provides compelling novel candidates for mediating paclitaxel resistance in breast cancer.

Experimental validation confirmed the interaction of PTX with AKT2. Pull‐down followed by western blotting revealed strong enrichment of AKT2 proteins in the PTX‐4 probe groups, as compared to the controls, despite equivalent amounts in input lysates (Figure 4e). Complementary CETSA experiments further demonstrated enhanced thermal stability of AKT2 upon PTX treatment, providing thermodynamic evidence of direct binding (Figure 4f). To further quantify the binding affinity and evaluate isoform selectivity, surface plasmon resonance (SPR) assays were performed using purified recombinant AKT1, AKT2, and AKT3 proteins. The results showed that PTX exhibited a strong binding affinity for AKT2 with a K_D_ value of 3.18 µM (Figure 4h ), while showing significantly weaker binding to AKT1 and AKT3. This direct biochemical evidence confirms that AKT2 is a high‐affinity binding partner of PTX. Beyond characterizing the binding affinity, we further investigated whether PTX binding directly modulates AKT2 enzymatic function, addressing a key question regarding its biochemical impact. Using an ADP‐Glo kinase assay, we demonstrated that PTX significantly inhibited the kinase activity of recombinant AKT2 in a dose‐dependent manner (Figure S6). This biochemical inhibition suggests that PTX acts as a direct modulator of AKT2 signaling, providing a functional link between physical binding and the underlying drug resistance.

**FIGURE 5:**
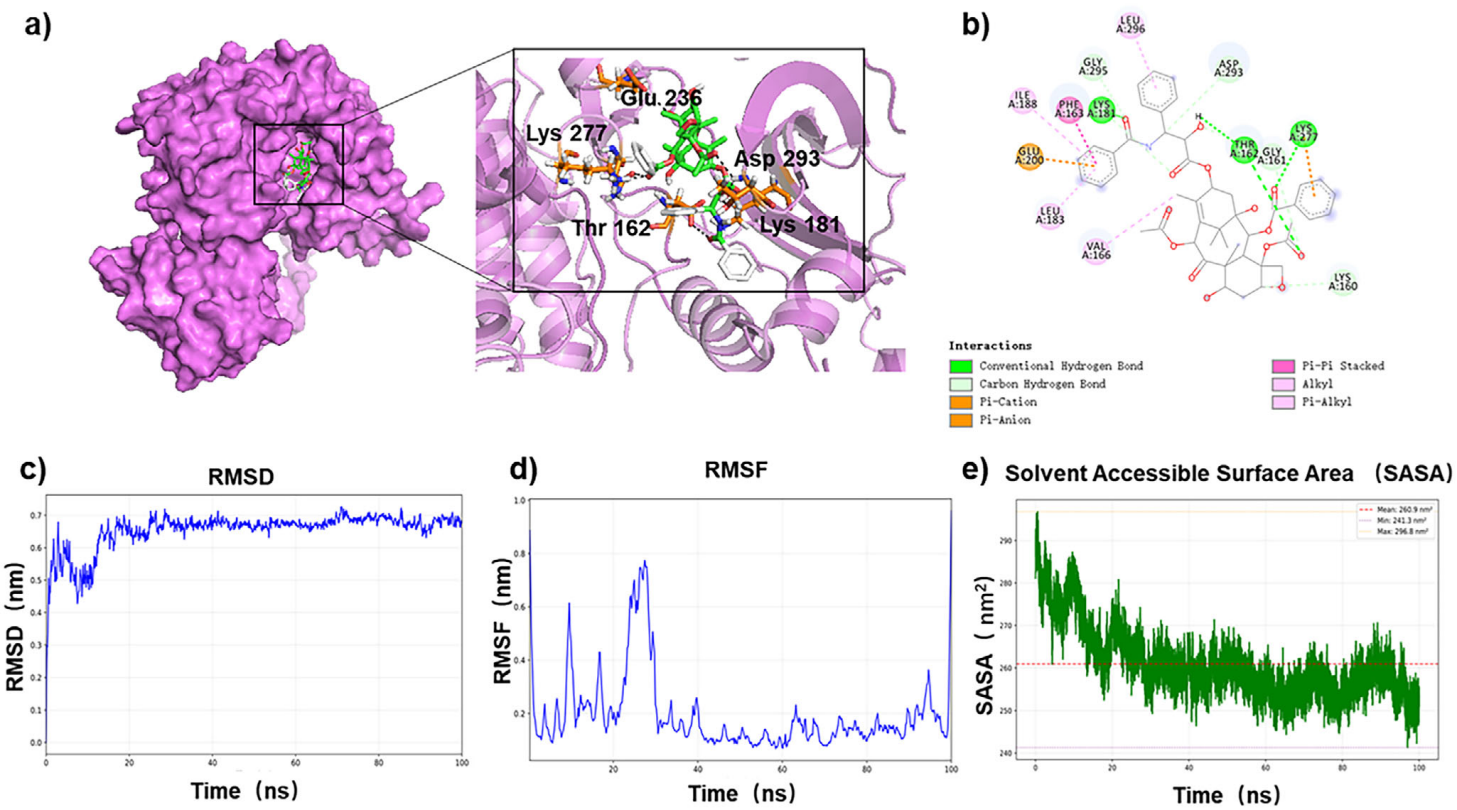
(a) PTX (green) binds to the catalytic pocket of AKT2, with the binding surface and details of the AKT2‐PTX complex in 3D imaging shown. Hydrogen bonds involved are indicated by black dashed lines. (b) Ligand‐protein interactions between PTX and AKT2 (2D diagram). (c–e) RMSD values, RMSF values, and SASA analysis of the AKT2‐PTX complex during 100 ns MD simulation.

To explore binding mechanisms in details, molecular docking studies were performed. PTX was predicted to form a stable conformation within the active pocket of AKT2, stabilized through hydrogen‐bond interactions with Lys181, Lys277, and Thr162 (Figure 5a,b). Molecular dynamics (MD) simulations further validated this interaction, showing stable complex behavior under physiological conditions as supported by RMSD, RMSF, and SASA analyses (Figure 5c–e). Collectively, these findings not only confirm the direct interaction of PTX with AKT2 but also highlight potential roles as novel molecular targets underlying PTX resistance in breast cancer.

### 2.6 AKT2 Knockdown and Inhibition Sensitize Resistant Breast Cancer Cells to PTX

**FIGURE 6:**
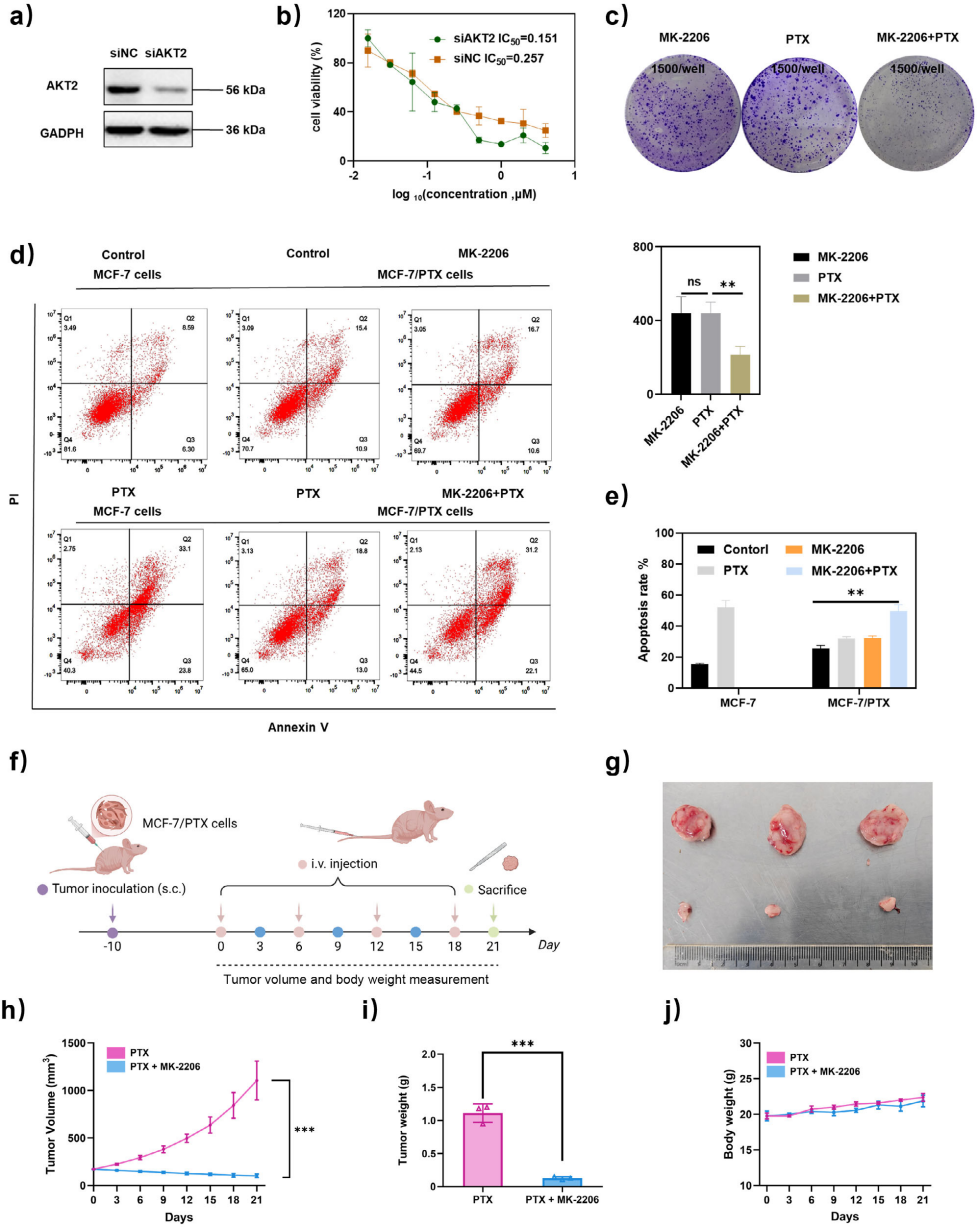
(a) Detection of AKT2 protein levels after siRNA knockdown. (b) Effect of AKT2 knockdown on the proliferation ability of MCF‐7/PTX cells. (c) Impact of the AKT2 inhibitor MK‐2206 on MCF‐7/PTX cell colonies and statistical analysis. (d, e) Representative flow cytometry plots and quantitative analysis of apoptosis. (**f)** Schematic illustration of the in vivo experimental design using MCF‐7/PTX xenografts. (**g)** Images of harvested tumors from PTX (up) and PTX + MK‐2206 groups(down) at day 21. (**h, i)** Tumor volume growth curves and final tumor weights. (**j)** Body weight monitoring of mice during the treatment period. To all the data above, ^*^ *p* < 0.05, ^**^ *p* < 0.01, and ^***^ *p* < 0.001. Differences between two groups were assessed by an unpaired t‐test, while comparisons among multiple groups were analyzed using one‐way ANOVA. Uncropped images of Figure 6a and the gels are provided in Figure S12.

Building on the identification of AKT2 as a potential PTX‐interacting protein, we next investigated its functional role in breast cancer drug resistance. AKT2 is well established as a central regulator of tumor cell survival and chemosensitivity across multiple cancer types, making it a promising therapeutic target in oncology [24]. To further elucidate the functional impact of the PTX‐AKT2 interaction on the classical mechanism of PTX, we conducted an in vitro tubulin polymerization assay. As expected, PTX significantly promoted tubulin assembly compared to the control group. Interestingly, the addition of recombinant human AKT2 protein attenuated PTX‐induced microtubule stabilization in a dose‐dependent manner (Figure S7), whereas the microtubule‐destabilizing agent nocodazole showed potent inhibition. These results suggest that the sequestration or interaction of PTX by upregulated AKT2 in resistant cells may impair the drug's primary ability to stabilize microtubules, thereby contributing to the development of PTX resistance. To directly assess its contribution to PTX resistance, AKT2 knockdown experiments were performed in MCF‐7/PTX cells. Transfection with AKT2‐specific siRNA effectively silenced its expression, as confirmed by western blot analysis 72 h post‐transfection (Figure 6a).

Functional evaluation revealed that downregulation of AKT2 significantly sensitized MCF‐7/PTX cells to PTX. Specifically, CCK‐8 assays showed a marked reduction in the IC_50_ of PTX in AKT2‐knockdown cells compared with negative controls (Figure 6b), indicating that suppression of AKT2 can reverse the resistant phenotype.

To further corroborate these findings, a pharmacological approach was employed using MK‐2206, a selective AKT2 inhibitor that prevents AKT2 phosphorylation at Ser473 [25, 26]. MCF‐7/PTX cells were treated with PTX alone, MK‐2206 alone, or the combination of both agents. The combination treatment exerted the strongest effects, significantly reducing colony formation (Figure 6c) and markedly increasing apoptosis, as quantified by flow cytometry (Figure 6d, e).

To further substantiate the involvement of the AKT2‐centered signaling axis in mediating drug resistance, we evaluated the effects of targeting upstream and parallel pathways in the MCF‐7/PTX model. Pharmacological inhibition of the upstream PI3K pathway (via Alpelisib) or the parallel MAPK pathway (via Ralimetinib) significantly resensitized the resistant MCF‐7 cells to PTX treatment (Figure S8). These findings demonstrate that the AKT2 signaling node is integrated into a broader network of resistance‐associated pathways, and its systematic modulation provides a unique approach to overcoming chemoresistance in breast cancer.

To assess the translational potential of AKT2 targeting, we established a xenograft mouse model using MCF‐7/PTX cells (Figure 6f). While PTX monotherapy failed to arrest tumor progression, the combination with MK‐2206 resulted in near‐complete tumor growth inhibition, as demonstrated by the representative tumor images (Figure 6g), tumor volume curves (Figure 6h), and final tumor weights (Figure 6i). Importantly, no significant body weight loss was observed in the combination group, suggesting favorable tolerability (Figure 6j). Collectively, these results establish AKT2 as a pivotal mediator of PTX resistance and a promising therapeutic target for sensitizing resistant breast cancer.

Together, these results demonstrate that AKT2 plays a pivotal role in mediating PTX resistance in breast cancer cells. Both genetic silencing and pharmacological inhibition effectively restored drug sensitivity, highlighting AKT2 as a promising therapeutic target for overcoming PTX resistance in breast cancer.

## 3 Discussions

The findings of this study highlight the potential of affinity‐based chemoproteomics in identifying biologically significant drug targets. We designed PTX affinity‐based probes and identified the resistance‐associated protein AKT2, providing novel insights into PTX‐relevant targets involved in drug resistance in MCF‐7 cells. The progression of breast cancer drug resistance involves the coordinated action of various protein molecules, receptors, and signaling pathways, the cascading effects of which have been extensively studied [18]. Notably, dysregulation of the phosphoinositide 3‐kinase (PI3K)/AKT/mammalian target of rapamycin (mTOR) pathway represents one of the major intracellular signaling cascades contributing to breast cancer drug resistance [18]. Most breast cancers exhibit alterations in the PI3K/AKT pathway, most commonly through somatic hotspot mutations in PIK3CA exons 9 and 20, which drive cell growth, proliferation, angiogenesis, and survival [19]. The serine/threonine kinase AKT (also known as protein kinase B) plays a central role in interconnected cellular signaling mechanisms regulating cell growth, apoptosis, and angiogenesis [27]. Consequently, the established importance of AKT in breast cancer and its implications for drug resistance have rendered it a highly sought‐after target for anticancer therapies. In this study, proteomic screening revealed upregulated AKT2 expression in MCF‐7 breast cancer cells with drug resistance. Both genetic knockdown experiments and pharmacological inhibition using MK‐2206 demonstrated that reduced AKT2 levels or activity could restore chemosensitivity. MK‐2206 administration has been investigated in multiple clinical trials beyond breast cancer, with in vitro evidence demonstrating its pro‐apoptotic efficacy against cancer cells [28]. These results collectively validate AKT2 as a potential therapeutic target for overcoming breast cancer drug resistance.

Previous studies have demonstrated that AKT2 is directly involved in malignant transformation and tumor dissemination [23]. Early work also revealed that siRNA‐mediated depletion of AKT2 led to reduced cell growth and invasiveness, confirming its potential role in tumorigenesis [29]. Furthermore, research by Petra Gener et al. showed that AKT2 inhibition not only affects “bulk” tumor cells but also impairs the survival of cancer stem cells (CSCs) under low‐attachment conditions. Additionally, AKT2 downregulation suppressed the invasive and transformative capacities of both bulk cells and CSC‐like populations, thereby reducing their tumorigenic potential and preventing tumor progression [30]. These findings collectively suggest AKT2 as a critical mediator of drug resistance.

This study identifies AKT2 as a central regulator of multidrug resistance in breast cancer and demonstrates that its inhibition can effectively restore chemosensitivity. Although activation of the PI3K/AKT/mTOR signaling cascade has been extensively implicated in breast cancer drug resistance [31], our findings provide mechanistic evidence that AKT2 is not merely a downstream effector but a pivotal driver of the PTX‐resistant phenotype. By employing an affinity‐based chemoproteomic approach, we established AKT2 upregulation as a hallmark of resistant cells and validated its functional necessity through genetic and pharmacological interventions.

These observations substantially extend prior reports that linked AKT2 to tumor progression, survival, and cancer stemness. While earlier studies suggested correlative associations, our results establish causality between AKT2 activity and drug resistance, thereby reframing AKT2 as a therapeutic target rather than a passive biomarker. Importantly, the ability of the selective allosteric inhibitor MK‐2206 to reverse resistance underscores the translational potential of AKT2 blockade in clinical settings where taxane failure remains a major obstacle.

From a therapeutic perspective, these findings align with a growing recognition that single‐agent chemotherapy is insufficient to overcome adaptive resistance mechanisms [32]. AKT2‐directed therapies, used in rational combination with standard chemotherapeutics, could represent a new paradigm for circumventing drug resistance in breast cancer. Such strategies may be particularly valuable in patients with relapsed or refractory disease, where resistance mechanisms severely limit available options.

Nonetheless, this work has limitations. Our analyses were mainly confined to in vitro models, and further in vivo studies are required to confirm the efficacy and tolerability of AKT2‐targeted interventions. Furthermore, whether AKT2 contributes to resistance against a broader spectrum of cytotoxic or targeted agents remains an open question. Elucidating the crosstalk between AKT2 and parallel signaling networks may also reveal synergistic vulnerabilities exploitable by multi‐target strategies.

The methodological success of the C─C bond‐linked probe design in this study offers a compelling blueprint for broader applications in chemical biology and drug discovery. Traditional affinity probes often rely on ester or amide linkages, which are susceptible to enzymatic hydrolysis by endogenous esterases or proteases within the native cellular environment. As demonstrated by the poor labeling performance of PTX‐1, such instability can significantly compromise the efficiency and specificity of target capture. In contrast, the robust intracellular stability provided by the C─C bond architecture in PTX‐4 allowed for the precise identification of physiologically relevant targets that might otherwise remain obscured. Looking forward, this design strategy constitutes a versatile platform that can be extended to other chemotherapeutic agents facing clinical resistance issues. By systematically profiling the drug interactome of diverse cytotoxic or targeted therapies through this high‐stability chemoproteomic approach, researchers can uncover unrecognized resistance mechanisms and identify novel synergistic targets. Such insights will provide a foundation for the rational design of next‐generation drugs and innovative combination regimens tailored to overcome adaptive resistance across various malignancies.

The identification of AKT2 as a functional mediator of PTX resistance highlights a tractable target for therapeutic intervention in breast cancer. Incorporating AKT2 inhibitors, such as MK‐2206, into taxane‐based regimens may offer a rational strategy to resensitize resistant tumors and extend the durability of response. These findings support the design of early‐phase clinical trials testing AKT2 blockade in combination therapy, particularly in patients with relapsed or refractory breast cancer where standard options are limited.

## 4 Conclusion

In summary, this study establishes a robust chemoproteomics‐based framework for systematically decoding the molecular interactome of drug resistance in breast cancer. By utilizing a rationally designed C─C bond‐linked affinity probe PTX‐4, we successfully identified AKT2 as a critical determinant of PTX resistance. Our multi‐level validation spanning from SPR kinetics and biochemical kinase assays to microtubule polymerization dynamics demonstrates that PTX directly binds to AKT2 and modulates AKT2 enzymatic activity, which interfere with classical drug target interactions. Functional investigations further reveal that AKT2 integrates into a broader signaling network, and its genetic or pharmacological inhibition effectively reverses the drug resistance phenotype. Most significantly, in vivo xenograft studies confirm that combining PTX with AKT2 blockade yields synergistic antitumor efficacy in mice. Collectively, these findings uncover a previously unrecognized mechanism of taxane resistance and provide a compelling foundation for developing AKT2‐targeted combination strategies to overcome clinical chemoresistance in breast cancer.

## 5 Experimental Section/Methods

### 5.1 General Methods

Except where otherwise stated, all analytically pure reagents were purchased from major vendors based in China and used directly without further purification. The ^1^H, ^13^C NMR spectra were taken on a Bruker nuclear magnetic resonance spectrometer (^1^H, 500 MHz; ^13^C, 101 MHz). Data for ^1^H NMR spectra are reported as follows: chemical shifts are reported as δ in units of parts per million (ppm) relative to tetramethylsilane (δ 0, s); multiplicities are reported as follows: s (singlet), d (doublet), t (triplet), q (quartet), dd (doublet of doublets), m (multiplet), or br (broadened); coupling constants are reported as a J value in Hertz (Hz); the number of protons (n) for a given resonance is indicated n H, and based on the spectral integration values.

### 5.2 Cell Lines and Cell Culture

MCF‐7 cells were cultured in DMEM supplemented with 10% fetal bovine serum (FBS) and 1% penicillin‐streptomycin. The cells were grown at 37°C in a 5% CO_2_ atmosphere until they reached the logarithmic growth phase, after which healthy and vigorously growing cells were selected for experimental use. Unless otherwise specified, all cell lines used in this study were cultured using this method.

### 5.3 Synthesis Procedures

Detailed synthetic routes, methods, and structural characterization of the compounds are listed in the supplementary materials.

### 5.4 Cell Proliferation Assay

When the cell confluency reached 80%–90%, the cells were digested to prepare a single‐cell suspension and seeded into a 96‐well plate, followed by incubation for 24 h. For the experimental groups, different concentrations of the probe and drug were added, with three replicates per concentration. The control group received culture medium containing an equal volume of DMSO, while the blank group consisted of culture medium without cells in the wells. After incubation for 48 h, the medium was removed, and 100 µL of CCK‐8 working solution was added to each well. The plate was further incubated for 1–2 h, and the optical density (OD) at 450 nm was measured using a microplate reader. Finally, the cell viability rate was calculated, and the half‐maximal inhibitory concentration (IC_50_) was determined using GraphPad Prism.

### 5.5 Cell Growth Curve

When MCF‐7 and MCF‐7/PTX cells reached approximately 80%–90% confluency, they were trypsinized to prepare single‐cell suspensions. Cell counts were performed, and cells were seeded into a 96‐well plate at a density of 1×10^3^ cells per well. The plate was then incubated in a humidified incubator at 37°C with 5% CO_2_. After 24 h, triplicate wells were used for each time point. To each well, 100 µL of cell culture medium containing 10% CCK‐8 reagent was added. The plate was measured at 450 nm wavelength using a microplate reader to determine the absorbance (OD value) of each well. This procedure was repeated daily at the same time for seven consecutive days. A growth curve was constructed with time (days) as the x‐axis and measured OD values as the y‐axis. The cell doubling time (T_d_) was calculated using the following formula: Td  =  T  ×  lg_2_ / lg(N_t_/N_0_). Where:T_d_ = cell doubling time,N_0_ = OD value when cells enter logarithmic growth phase,N_t_ = OD value after t hours of culture,T = time elapsed from N_0_ to N_t_.

### 5.6 Colony Formation Assay

When cells reached approximately 80%–90% confluency, they were trypsinized to prepare single‐cell suspensions. Cells were seeded into 6‐well plates at densities of 500 cells/well and 1500 cells/well, with 3 replicate wells. The plates were incubated in a humidified incubator at 37°C with 5% CO_2_. After 24 h, the medium was replaced with fresh medium containing PTX (100 nmol/L), and cells were cultured for an additional 12 h. Following this treatment period, the medium was aspirated, and each well was washed twice with PBS. Complete culture medium was then added. The medium was changed every 2 days. After 10 days, the medium was aspirated, and each well was washed twice with PBS. Then, 1 mL of 4% paraformaldehyde was added to each well for fixation (30 min). Each well was washed twice with PBS. Subsequently, 1 mL of crystal violet solution was added to each well for staining at room temperature (10 min). Excess crystal violet was removed by PBS washing. Cell counting was performed using FIJI software. The colony formation efficiency was calculated using the formula: Colony formation efficiency  =  (number of colonies/number of seeded cells)  ×  100%.

### 5.7 Cell Resistance Index

When the cell density reached approximately 80%–90%, a cell suspension was prepared. The cells were seeded in 6‐well plates at a density of 2500 cells per well, with three replicate wells per group, and placed in a 37°C, 5% CO_2_ incubator. After 24 h, the original medium was aspirated and replaced with 100 µL per well of medium containing PTX, with three replicate wells set up. A drug‐free control group was also established, and a blank group consisting of complete medium without cells was included. The plates were then returned to the incubator. After 48 h of incubation, the old medium was removed, and 100 µL of cell medium containing 10% CCK‐8 was added to each well. The plates were then incubated for 1–2 h. The OD value of each well was measured at a wavelength of 450 nm using a microplate reader. Cell viability was calculated, and the IC_50_ value of the drug was determined using GraphPad Prism software. Following the same method, the IC_50_ values of conventional chemotherapeutic drugs—doxorubicin (ADM), gemcitabine (GEM), cisplatin (DDP), and 7‐ethyl‐10‐hydroxycamptothecin (SN‐38)—were measured in both MCF‐7 and MCF‐7/PTX cells. The corresponding resistance index (RI = IC_50_ (MCF‐7/PTX) / IC_50_ (MCF‐7)) was then calculated.

### 5.8 Live Cell Labeling

Cells were seeded in 12‐well plates and cultured until reaching 80%–90% confluency. The experimental group was incubated with probe‐containing medium, while the control group received medium containing DMSO. After removing the medium, cells were washed twice with PBS. Subsequently, cells were placed on ice and exposed to UV irradiation (365 nm). PBS was aspirated, and cell lysis buffer was added to each well. Cells were subjected to repeated freeze‐thaw cycles, gently scraped, and the lysate was transferred to 2 mL centrifuge tubes. For the click chemistry reaction (CuAAC), 10 mM THPTA, 100 mM TCEP, and 100 mM CuSO_4_·5H_2_O solutions were sequentially added and mixed thoroughly. The solution was monitored for a color change to green, after which 10 mM TER‐N_3_ solution was added at a 1:1:1:1 volume ratio. The click reaction mixture was then added to the cell lysate and incubated on a thermomixer at 25°C with 800 rpm shaking for 2 h. After the reaction, 1 mL of pre‐chilled acetone was added to each tube, followed by incubation at −20°C for 2 h. Samples were then centrifuged at 4°C, 12 000 rpm for 10 min. The supernatant was discarded, and residual acetone was completely evaporated. Subsequently, 200 µL of pre‐chilled methanol was added to each tube, followed by sonication to dissolve the precipitate. Tubes were stored at −20°C for 2 h, centrifuged again, and the supernatant was carefully removed. Tubes were inverted to allow complete methanol evaporation. For subsequent steps, 2X loading buffer was added, and samples were sonicated to ensure complete protein dissolution. Finally, samples were heated at 95°C for 10 min and then subjected to gel electrophoresis.

### 5.9 Competitive Labeling

Cells were seeded in 12‐well plates and cultured until reaching 80%–90% confluency. For the competition group, cells were first incubated with PTX‐containing DMEM medium for 2 h. Subsequently, both the competition group and experimental group were treated with probe‐containing medium, while the control group received DMSO‐containing medium. The subsequent experimental procedures followed the standard protocol for live‐cell in situ labeling.

### 5.10 Pull‐Down Experiment

Cells were seeded in 10 cm culture dishes and cultured until reaching 80%–90% confluency. The control group was treated with DMSO, while the experimental group was incubated with the probe. Following incubation, the medium was aspirated, and cells were washed twice with PBS. Cells were placed on ice and irradiated with UV light (365 nm) for 1 h. Subsequently, 1 mL of cell lysis buffer was added to each dish, followed by three freeze‐thaw cycles. After centrifugation (12 000 rpm, 4°C, 10 min), the supernatant was collected for subsequent experiments. Protein concentration was determined using the BCA assay and adjusted to equal concentrations with PBS. The click reaction was performed by sequentially adding pre‐mixed reagents: 10 mM THPTA, 100 mM TCEP, 100 mM CuSO_4_·5H_2_O, and 10 mM TER‐Biotin‐N_3_ (10:10:10:1). The reaction mixture was incubated at 25°C with shaking (800 rpm) for 2 h. Ice‐cold acetone was added to each tube, followed by incubation at −20°C for 2 h. After centrifugation (12 000 rpm, 4°C, 10 min), the supernatant was discarded, and residual acetone was completely evaporated. Subsequently, 1 mL of methanol was added to each tube, followed by sonication and incubation at ‐20°C for 2 h. The samples were then centrifuged (12 000 rpm, 4°C, 10 min), and the supernatant was discarded. This methanol washing procedure was repeated three times. The protein pellet was resuspended in 800 µL of PBS containing 1 M urea and 1% SDS, followed by sonication and centrifugation (12 000 rpm, room temperature, 10 min). A portion of the supernatant was analyzed by SDS‐PAGE to verify fluorescence signal presence. If fluorescence was detected, the remaining sample was processed for further experiments. Streptavidin magnetic beads were pre‐washed three times with each of the following solutions: PBS, 1 M urea, and 1 M urea containing 1% SDS. The cell lysate was then incubated with the pre‐washed beads at room temperature for 2 h with gentle rotation. Following incubation, the supernatant was removed, and the beads were washed sequentially with: 6 M urea, PBS, and deionized water. One‐tenth of the washed beads were resuspended in 2X protein loading buffer, boiled, and analyzed by SDS‐PAGE to confirm fluorescence. If fluorescent protein bands were observed, the remaining beads were resuspended in ddH_2_O and submitted for mass spectrometry analysis.

### 5.11 Western Blot

After enriching proteins with streptavidin magnetic beads, add 2X protein loading buffer and heat the samples at 95°C for 15 min, followed by SDS‐PAGE electrophoresis. Prepare a PVDF membrane by activating it in methanol for 15–30 s. Assemble the gel and PVDF membrane in a “sandwich” configuration, ensuring no bubbles are trapped. Transfer the proteins at 70 V for 1.5 h. Place the PVDF membrane in an incubation box and block it with 5% skim milk for 2 h. After blocking, wash the membrane three times with TBST (10 min each). Add the prepared primary antibody solution and incubate at room temperature with shaking for 1.5 h. After incubation, wash the membrane three times with TBST (15 min each). Add the prepared secondary antibody solution and incubate at room temperature for 1 h. After incubation, wash the membrane three times with TBST (15 min each). Add ECL substrate and perform chemiluminescent detection using an iBright imaging system. Analyze the band intensities using ImageJ software by calculating the ratio of the target protein grayscale value to the internal reference protein grayscale value. Normalize the data relative to the control group and perform statistical analysis using GraphPad Prism software for significance testing.

### 5.12 Cellular Thermal Shift Assay (CETSA)

Cells were seeded in 10 cm cell culture dishes at a density of 80%–90% and incubated with PTX (100 µM) or DMSO at 37°C under 5% CO_2_ for 3 h. After washing with PBS containing protease inhibitors, the cells were collected and divided into 10 groups. The samples were heated at gradient temperatures (54°C–81°C) for 3 min each, then cooled on ice. After all heating steps, the samples were subjected to freeze‐thaw lysis in liquid nitrogen, repeated three times, to obtain cell lysates. The lysates were centrifuged (15 000 × g, 15 min, 4°C), and the supernatants were collected. Then, 5 X loading buffer was added, and the samples were heated at 95°C for 10 min before proceeding with Western blot analysis.

### 5.13 Small Interfering RNA (siRNA) Transfection

The small interfering RNA (siRNA) targeting AKT2 and the negative control (NC) were synthesized by Tsingke Biotechnology. The sequence of si‐AKT2 was GUGCUGGAGGACAAUGACU. Transfection was performed using Lipofectamine 2000 according to the manufacturer's instructions. Cells were seeded in 24‐well plates, divided into the NC group and the siAKT2 group, with three replicates per group, at a density of approximately 50%–60%. The diluted transfection reagent was mixed with siRNA at a concentration of 50 nM and incubated at room temperature for 20 min. The transfection complexes were then added to the cell culture medium. After 6 h, the medium was replaced with fresh medium. The cells were further cultured in the incubator, and protein expression was assessed by Western blot 72 h post‐transfection. Cells were seeded in 6‐well plates with three replicates per group. First, they were treated with medium containing the MK‐2206 inhibitor for 24 h, followed by replacement with medium containing PTX (0.2 µM) for 12 h. The cells were then washed twice with PBS, trypsinized, and centrifuged. The cell pellets were washed three times with pre‐cooled PBS. After collecting the cell pellets, 100 µL of 1X Annexin V Binding Buffer was added to each tube to resuspend the cells according to the manufacturer's instructions. Next, 5 µL of Annexin V‐APC and 5 µL of PI Staining Solution were added to each tube and gently mixed. The samples were incubated at room temperature in the dark for 15 min. Finally, 400 µL of 1X Binding Buffer was added to each tube and gently mixed. Apoptosis was analyzed by flow cytometry within 1 h.

### 5.14 Molecular Docking and Molecular Dynamics Simulation

Molecular Docking and Molecular Dynamics Simulation: Molecular docking was performed using AutoDock to investigate the binding mode of PTX with the predicted structure of human AKT2 (AlphaFold model AF‐P31751‐F1‐v6). The grid box was centered on the active pocket covering key residues, including Lys181, Thr162, Glu236, Lys277, and Asp293. The optimal docking conformation was selected as the starting structure for molecular dynamics (MD) simulations, which were executed for 100 ns using GROMACS under the AMBER99SB‐ILDN force field. The system was solvated in a TIP3P water box, neutralized with 0.15 M NaCl, and equilibrated under NVT and NPT ensembles prior to the production run. System stability and interaction dynamics were evaluated through Root‐Mean‐Square Deviation (RMSD), Root‐Mean‐Square Fluctuation (RMSF), and Solvent Accessible Surface Area (SASA) analyses.

### 5.15 LC‐MS/MS Analysis and Proteomic Data

Protein samples enriched on streptavidin magnetic beads were washed, reduced, alkylated, and digested with trypsin on the beads. The resulting peptides were desalted and analyzed using an Orbitrap Fusion Lumos mass spectrometer coupled with a Thermo Fisher Scientific nano‐liquid chromatography system. The peptides were loaded onto a C18 reversed‐phase column (75 µm × 25 cm, 1.9 µm particle size) and separated at a flow rate of 300 nL/min. A linear gradient elution was performed between mobile phase B (80% acetonitrile solution of 0.1% formic acid) and mobile phase A (0.1% aqueous solution of formic acid) for 90 min: the proportion of mobile phase B increased from 5% to 30% over 70 min; then from 30% to 45% over 10 min; finally from 45% to 95% over 5 min; followed by elution with 95% mobile phase B for 5 min. MS1 scan data were acquired on an Orbitrap mass spectrometer at a resolution of 120 000 m/z. The mass spectrometry range was 350–1600 (m/z). Precursor ions were separated using a quadrupole mass spectrometer with an isolation window of 0.7 Da and fragmented by high‐energy collisional dissociation (HCD) at a normalized collision energy of 34%. MS2 spectra were recorded on an Orbitrap mass spectrometer at a resolution of 50 000. Raw data files were processed using Thermo Proteome Discoverer 2.5 software with a built‐in Sequest search engine and reference to the UniProt human database. A strict 1% false discovery rate (FDR) threshold was applied for identification and subsequent qualitative/quantitative analysis at both the peptide and protein levels.

### 5.16 Animal Experiment

Female BALB/c nude mice (4–6 weeks old) were purchased from Hangsi‐bio (Hangzhou, China) and acclimated in a specific pathogen‐free (SPF) environment under a 12‐h light/dark cycle with ad libitum access to food and water. All animal experimental procedures were strictly approved by the Experimental Animal Welfare and Ethics Review Committee of Zhejiang University of Technology (Approval No. MGS20260107002) prior to initialization. To establish the tumor xenograft model, MCF‐7/PTX cells were trypsinized, washed, and resuspended in ice‐cold phosphate‐buffered saline (PBS). A total of 3 × 10^6^ cells in 50 uL of PBS were thoroughly blended with 50 uL of Matrigel (Novoprotein,Suzhou,China), yielding a final inoculum volume of 100 uL per mouse, which was subsequently inoculated subcutaneously into the right flank of each mouse. Once the average tumor volume reached approximately 100 mm^3^, the mice were randomly allocated into two cohorts (*n* = 3 per group): the PTX monotherapy group and the PTX + MK‐2206 combination therapy group. All therapeutic regimens were administered via tail vein injections. In the monotherapy group, PTX was delivered at a dose of 10 mg/kg every 6 days; the combination group received an identical dose of PTX (10 mg/kg) supplemented with MK‐2206 (20 mg/kg) on the same schedule. Tumor volume (V) and murine body weight were monitored every 3 days using digital calipers, where V was calculated using the standard formula: V = (Length×Width^2^)/2. On day 21, the mice were humanely euthanized, and the xenograft tumors were harvested and weighed for further evaluation.

### 5.17 Statistical Analysis

The reported values served as averages of at least three biological replicates and represent independent biological experiments. All data are presented as mean ± standard deviation (SD) and analyzed using GraphPad Prism 9.0 (GraphPad Software). Two groups were analyzed by T test, and more than two groups were calculated by One/Two‐way ANOVA. The data were considered significant, when ^***^ *p* < 0.001; ^**^ *p* < 0.01; ^*^ *p* < 0.05; ns, no significant.

## Author Contributions

K.W., Y.Y., Y.H., and Q.Z. conceptualized the research. K.W., Y.Y., W.S., X.W., and H.Z. contributed resources. Y.H. and Q.Z. acquired funding and supervised the project. Y.Y. and Q.Z. developed the methodology. K.W. and Y.Y. performed experiments and analyzed the data. K.W. conducted software applications and calculations. Q.Z. maintained data curation and managed project administration. K.W. and Y.Y. wrote the original draft of the manuscript. K.W., W.S., X.Y., X.W., H.Z., Y.H., and Q.Z. reviewed and edited the manuscript. All authors have given approval to the final version of the manuscript.

## Conflicts of Interest

The authors declare no conflicts of interest.

## Supporting information




**Supporting File**: advs76417‐sup‐0001‐SuppMat.docx.

## Data Availability

All processed proteomic identification and label‐free quantification datasets generated and analyzed during this study have been deposited in the Figshare repository and are publicly accessible via the DOI identifierhttps://doi.org/10.6084/m9.figshare.32759868. All other sequence data, characterization profiles, and supporting experimental findings are provided within the Supplementary Materials of this article.
