# Supplementary material for: Chemoproteomic Identification of AKT2 as a Paclitaxel‐Binding Protein via C─C Bond‐Linked Probe PTX‐4 in Paclitaxel‐Resistant Breast Cancer
Source: Adv Sci (Weinh). 2026 Jul 30;13(54):e20089. doi: 10.1002/advs.202520089 (PMC13423490; doi:10.1002/advs.202520089)
Supplement: Supplementary file 1 [file ADVS-13-e20089-s001.docx]

**SUPPORTING INFORMATION**

**Chemoproteomic Identification of AKT2 as A Paclitaxel-Binding Protein via C-C Bond-Linked Probe PTX-4 in Paclitaxel-Resistant Breast Cancer**

Kai Wang^1^, Yuqing Yuan^1^, Wei Shen^2,^* , Xiuxiu Yang^1^, Xiaokang Wu^2^, Haihua Zhou^2^, Yi Hu^3,^*, Qing Zhu^1,,^*

^1^State Key Laboratory of Green Chemical Synthesis and Conversion, Hangzhou 310014, China. Email: *zhuq@zjut.edu.cn*

^2^Department of Surgery, Affiliated Jinhua Hospital, Zhejiang University School of Medicine, Jinhua 321000, China. Email: [*shenweiri@163.com*](mailto:shenweiri@163.com)

^3^State Key Laboratory of Complex, Severe, and Rare Diseases, Biomedical Engineering Facility of National Infrastructures for Translational Medicine, Institute of Clinical Medicine, Peking Union Medical College Hospital, Chinese Academy of Medical Sciences and Peking Union Medical College, Beijing 100730, China. Email: *huyi@u.nus.edu*

**Table of contents**

[Synthesis Procedures	2](#_Toc234171872)

[Supplementary Figures	7](#_Toc234171873)

[Table S1 PTX target proteins identified by PTX-4 probe in MCF-7 cells	12](#_Toc234171874)

[Table S2 PTX target proteins identified by PTX-4 probe in MCF-7/PTX cells	17](#_Toc234171875)

[NMR Spectrums	21](#_Toc234171876)

# Synthesis Procedures

Synthesis of PTX-1 probe

*Synthesis of compound 1a.* Paclitaxel (PTX, 500 mg, 0.6 mmol) and imidazole (199.43 mg, 3.0 mmol) were dissolved in anhydrous dichloromethane. TESCl (150 μL, 0.9 mmol) was slowly added to the mixture and stirred continuously at room temperature. Monitored the reaction progress by TLC. The reaction solution was extracted with water and dichloromethane. The organic layer was dried over anhydrous Na_2_SO_4_. Removed the organic solvent by rotary evaporation. The crude product was purified by column chromatography (EtOAc:PE = 1:2) to obtain compound 1a (430 mg), yield 90%.^1^H NMR(500 MHz，Chloroform-d)δ 8.16–8.13 (m, 2H), 7.77 – 7.74 (m, 2H), 7.64 – 7.59 (m, 1H), 7.55 – 7.48 (m, 5H), 7.44 – 7.39 (m, 4H), 7.38 – 7.32 (m, 3H), 7.14 (d, J= 8.9 Hz, 1H), 6.31 (s, 1H), 6.29 – 6.26 (m, 1H), 5.75 – 5.69 (m, 2H), 4.99 (dd, J = 9.7, 2.3 Hz, 1H), 4.71 (d, J = 2.1 Hz, 1H), 4.45 (dd, J = 11.0, 6.6 Hz, 1H), 4.34 (d, J = 8.4 Hz, 1H), 4.23 (dd, J = 8.4, 1.1 Hz, 1H), 3.85 – 3.81 (m, 1H), 2.61 – 2.56 (m, 1H), 2.55 (s, 3H), 2.45 – 2.37 (m, 1H), 2.24 (s, 3H), 2.19 (dd, J = 15.3, 8.7 Hz, 1H), 1.92 (d, J = 9.9 Hz, 3H), 1.88 (d, J = 2.4 Hz, 1H), 1.70 (s, 3H), 1.26 (s, 3H), 1.15 (s, 3H), 0.84 (t, J = 7.9 Hz, 9H), 0.60 – 0.39 (m, 6H). ^13^C NMR (126 MHz, Chloroform-d) δ 171.55 , 171.30 , 167.04 , 166.97 , 142.45 , 133.67 , 132.94 , 131.80 , 130.22 , 129.16 , 128.77 , 128.72 , 128.70 , 128.03 , 127.04 , 126.46 , 84.45 , 81.14 , 79.07 , 76.48 , 75.56 , 75.09 , 74.86 , 72.11 , 71.49 , 58.50 , 55.71 , 45.51 , 43.25 , 35.81 , 35.56 , 26.76 , 23.00 , 22.28 , 20.85 , 14.85 , 9.63 , 6.53 , 4.37 .

*Synthesis of compound 1b.* In a 100 mL round-bottom flask under an inert nitrogen atmosphere, compound 1a (200 mg, 0.2 mmol), DCC (63.98 mg, 0.3 mmol), and DMAP (12.62 mg, 0.1 mmol) were dissolved in 10 mL DMF. 5-hexynoic acid (34.7 mg, 0.3 mmol) was slowly added dropwise, and the reaction mixture was stirred at room temperature for 12 h. The consumption of 1a was monitored by TLC. The mixture was sequentially extracted with EtOAc. The organic layer was washed with aqueous solutions of saturated NaHCO_3_ and brine, dried over anhydrous Na_2_SO_4_, and filtered to remove the drying agent. The crude product was purified by column chromatography (EtOAc:PE = 1:3) to afford white solid compound 1b (140 mg), yield 60%.^1^H NMR (500 MHz, Chloroform-d) δ 8.16 – 8.13 (m, 2H), 7.79 – 7.75 (m, 2H), 7.65 – 7.61 (m, 1H), 7.56 – 7.49 (m, 3H), 7.45 – 7.39 (m, 4H), 7.37 – 7.33 (m, 3H), 7.15 (d, J = 8.9 Hz, 1H), 6.30 (s, 1H), 6.26 (td, J = 9.2, 1.7 Hz, 1H), 5.74 – 5.70 (m, 2H), 5.63 (dd, J = 10.6, 7.0 Hz, 1H), 4.99 (dd, J = 9.6, 1.9 Hz, 1H), 4.71 (d, J = 2.0 Hz, 1H), 4.36 (d, J = 8.5 Hz, 1H), 4.25 – 4.21 (m, 1H), 3.99 (d, J = 6.9 Hz, 1H), 2.61 (ddd, J = 14.3, 9.6, 7.0 Hz, 1H), 2.56 (s, 3H), 2.51 – 2.45 (m, 1H), 2.45 – 2.37 (m, 2H), 2.26 (tt, J = 7.0, 2.7 Hz, 2H), 2.23 – 2.19 (m, 1H), 2.17 (s, 3H), 2.00 (d, J = 1.4 Hz, 3H), 1.96 (t, J = 2.6 Hz, 1H), 1.90 – 1.86 (m, 1H), 1.86 – 1.82 (m, 5H), 1.23 (s, 3H), 1.18 (s, 3H), 0.83 (t, J = 7.9 Hz, 9H), 0.52 – 0.39 (m, 6H).

Synthesis of compound PTX-1. Compound 1b (300 mg, 0.3 mmol) was dissolved in anhydrous THF (10 mL), and a solution of TBAF (1.0 M in THF, 89 μL, 0.3 mmol) was slowly added dropwise. The mixture was stirred at room temperature for 1 h, and the reaction was monitored by TLC. The reaction was quenched by dilution with water, and the mixture was extracted with dichloromethane. The organic layer was dried over anhydrous Na_2_SO_4_, and concentrated. The crude product was purified by column chromatography (EtOAc:PE = 1:2) to afford white solid PTX-1 (175 mg), yield 67%.^1^H NMR (400 MHz, Chloroform-d) δ 8.13 – 8.06 (m, 2H), 7.78 – 7.70 (m, 2H), 7.63 – 7.56 (m, 1H), 7.52 – 7.45 (m, 5H), 7.39 (td, J = 7.4, 1.9 Hz, 4H), 7.36 – 7.31 (m, 1H), 7.17 (d, J = 9.0 Hz, 1H), 6.22 (s, 1H), 6.19 – 6.12 (m, 1H), 5.79 (dd, J = 9.0, 2.6 Hz, 1H), 5.66 (d, J = 6.9 Hz, 1H), 5.55 (dd, J = 10.5, 7.2 Hz, 1H), 4.93 (dd, J = 9.5, 1.8 Hz, 1H), 4.82 – 4.76 (m, 1H), 4.30 (d, J = 8.5 Hz, 1H), 4.18 (d, J = 8.4 Hz, 1H), 3.90 (d, J = 6.9 Hz, 1H), 3.85 (s, 1H), 2.61 – 2.38 (m, 3H), 2.36 (s, 3H), 2.34 – 2.29 (m, 2H), 2.25 – 2.20 (m, 2H), 2.15 (s, 3H), 2.01 – 1.92 (m, 2H), 1.84 – 1.78 (m, 8H), 1.44 – 1.21 (m, 4H), 1.17 (d, J = 11.1 Hz, 6H). ^13^C NMR (126 MHz, Chloroform-d) δ 172.42 , 172.20 , 168.89 , 167.11 , 140.35 , 138.03 , 133.76 , 133.65 , 131.93 , 130.15 , 129.09 , 128.95 , 128.72 , 128.69 , 128.28 , 127.06 , 83.89 , 83.63 , 81.02 , 78.44 , 76.43 , 75.21 , 74.33 , 73.22 , 72.09 , 71.30 , 68.87 , 56.16 , 54.94 , 46.93 , 43.22 , 35.56 , 33.41 , 32.86 , 26.52 , 23.20 , 22.54 , 20.83 , 20.74 , 17.80 , 14.61 , 10.83 .

Baccatin III (500 mg, 0.85 mmol) was dissolved in 20 mL THF, followed by the addition of imidazole (290 mg, 4.2 mmol). TESCl (0.3 mL, 1.7 mmol) was then added dropwise at room temperature, and the reaction mixture was stirred for 0.5 h. The reaction mixture was transferred to a separatory funnel and extracted with water and EtOAc. The organic layer was collected, dried over anhydrous Na_2_SO_4_, and concentrated. The crude product was purified by column chromatography (EtOAc:PE = 1:2) to obtain white solid Baccatin III-1 (470 mg), yield 95%.^1^H NMR (500 MHz, Chloroform-d) δ 8.14 – 8.08 (m, 2H), 7.63 – 7.58 (m, 1H), 7.51 – 7.45 (m, 2H), 6.47 (s, 1H), 5.64 (d, J = 7.1 Hz, 1H), 4.97 (dd, J = 9.7, 2.0 Hz, 1H), 4.84 (ddd, J = 9.0, 7.2, 1.7 Hz, 1H), 4.50 (dd, J = 10.5, 6.7 Hz, 1H), 4.34 – 4.28 (m, 1H), 4.15 (dd, J = 8.3, 1.1 Hz, 1H), 3.89 (dd, J = 7.1, 1.0 Hz, 1H), 2.54 (ddd, J = 14.3, 9.6, 6.7 Hz, 1H), 2.29 (s, 3H), 2.29 – 2.24 (m, 2H), 2.19 (d, J = 3.9 Hz, 6H), 1.88 (ddd, J = 14.3, 10.5, 2.1 Hz, 1H), 1.69 (s, 3H), 1.26 (d, J = 1.7 Hz, 1H), 1.20 (s, 3H), 1.04 (s, 3H), 0.93 (t, J = 7.9 Hz, 9H), 0.59 (qd, J = 7.9, 4.5 Hz, 6H). ^13^C NMR (126 MHz, Chloroform-d) δ 202.21 , 170.73 , 167.09 , 143.99 , 133.61 , 132.65 , 130.09 , 128.58 , 84.22 , 80.84 , 78.73 , 76.52 , 75.79 , 74.73 , 72.35 , 67.92 , 58.65 , 47.26 , 42.78 , 38.27 , 37.24 , 26.81 , 22.68 , 20.96 , 20.09 , 14.96 , 9.95 , 6.75 , 5.28 .

Synthesis of PTX-2 probe

*Synthesis of compound 2a.* (2R,3S)-Methyl 3-amino-2-hydroxy-3-phenylpropanoate (500 mg, 2.5 mmol), 4-ethynylbenzoic acid (450 mg, 3.0 mmol), EDCI (736.5 mg, 3.8 mmol), and HOBT (519 mg, 3.8 mmol) were sequentially added to a 100 mL round-bottom flask. The mixture was dissolved in anhydrous DMF (30 mL), followed by the slow addition of DIEA (1.3 mL, 7.6 mmol). The reaction was stirred at room temperature for 5 h. After completion, the reaction mixture was diluted with EtOAc The organic layer was successively washed with dilute hydrochloric acid and saturated NaHCO_3_, followed by saturated brine. The organic phase was then dried over anhydrous Na_2_SO_4_ and concentrated. The crude product was purified by silica gel column chromatography (EtOAc:PE = 1:1) to afford compound 2a (450 mg), yield 80%.^1^H NMR (400 MHz, DMSO-d6) δ 8.84 (d, J = 8.9 Hz, 1H), 7.87 (d, J = 8.3 Hz, 2H), 7.61 (d, J = 8.0 Hz, 2H), 7.41 (d, J = 7.2 Hz, 2H), 7.35 – 7.30 (m, 2H), 7.28 – 7.23 (m, 1H), 5.88 (d, J = 7.8 Hz, 1H), 5.42 (dd, J = 8.9, 5.5 Hz, 1H), 4.50 (s, 1H), 4.40 (s, 1H). ^13^C NMR (101 MHz, DMSO-d6) δ 172.39 , 165.44 , 139.54 , 134.42 , 131.67 , 129.90 – 125.79 (m), 124.59 , 82.92 (d, J = 4.4 Hz), 73.67 , 56.22 , 51.61 , 39.52 (dp, J = 41.8, 21.0 Hz).

*Synthesis of compound 2b.* Compound 2a (300 mg, 0.9 mmol) and pyridinium p-toluenesulfonate (PPTS) (23.3 mg, 0.1 mmol) were dissolved in 20 mL anhydrous toluene under an inert nitrogen atmosphere. 4-Methoxybenzaldehyde dimethyl acetal (238 μL, 1.5 mmol) was then added dropwise. The reaction mixture was refluxed for 1 h, after which the temperature was allowed to return to room temperature.

The reaction was quenched by extraction with EtOAc, and the organic layer was successively washed with saturated NaHCO_3_ and saturated brine. The organic phase was dried over anhydrous Na_2_SO_4_ and concentrated. The crude product was purified by silica gel column chromatography (EtOAc:PE = 1:5) to afford compound 2b (200 mg), yield 70%.^1^H NMR (500 MHz, DMSO-d6) δ 7.40 (d, J = 8.0 Hz, 3H), 7.35 (d, J = 7.8 Hz, 4H), 7.24 (d, J = 7.9 Hz, 4H), 6.91 (s, 2H), 6.59 (s, 1H), 5.49 – 5.21 (m, 1H), 5.02 (s, 1H), 4.34 (s, 1H), 3.78 (s, 3H), 3.76 (s, 3H).^13^C NMR (126 MHz, DMSO-d6) δ 170.25 , 160.00 , 139.47 , 136.39 , 132.08 , 129.75 , 129.00 , 128.38 , 127.21 , 124.11 , 113.97 , 90.71 , 83.08 , 55.60 , 53.02 .

*Synthesis of compound 2c.* Compound 2b (100 mg, 0.2 mmol) was dissolved in 10 mL MeOH, and LiOH·H₂O (11 mg, 0.2 mmol) was slowly added. The reaction mixture was stirred at room temperature for 1 h, with the progress monitored by TLC. MeOH was removed under reduced pressure. The mixture was diluted with water, acidified with dilute HCl, and extracted sequentially with EtOAc. The organic phase was dried over anhydrous Na₂SO₄ and concentrated to afford crude compound 2c for the next step.

*Synthesis of compound 2d.* Baccatin III-1 (600 mg, 0.9 mmol), 2c (732 mg, 1.8 mmol), DCC (265 mg, 1.3 mmol), and DMAP (52 mg, 0.4 mmol) were sequentially added to a 50 mL round-bottom flask. The mixture was dissolved in anhydrous toluene (6 mL) under an inert nitrogen atmosphere and stirred at room temperature for 5 h. The reaction mixture was diluted with EtOAc and sequentially extracted with water and saturated aqueous NaHCO₃ solution, followed by washing with saturated brine. The organic layers were dried over anhydrous Na_2_SO_4_, and the solvent was removed under reduced pressure by rotary evaporation. The crude product was purified by column chromatography (EtOAc:PE = 1:3) to afford white solid 2d (585 mg), yield 80%.^1^H NMR (500 MHz, DMSO-d6) δ 8.04 – 7.98 (m, 2H), 7.71 – 7.64 (m, 1H), 7.56 (t, J = 7.7 Hz, 2H), 7.43 – 7.32 (m, 7H), 7.28 (s, 2H), 6.98 (d, J = 18.5 Hz, 2H), 6.37 (s, 1H), 6.15 (t, J = 9.1 Hz, 1H), 5.52 (d, J = 7.0 Hz, 1H), 5.11 (s, 1H), 4.98 (s, 1H), 4.92 (d, J = 9.5 Hz, 1H), 4.43 (dd, J = 10.4, 6.9 Hz, 1H), 4.34 (s, 1H), 4.04 (s, 2H), 3.79 (s, 3H), 3.76 (d, J = 7.1 Hz, 1H), 2.39 – 2.31 (m, 1H), 2.24 (dd, J = 15.2, 8.8 Hz, 1H), 2.13 (d, J = 13.2 Hz, 6H), 2.07 – 2.03 (m, 3H), 1.70 – 1.61 (m, 1H), 1.56 (s, 3H), 1.24 (s, 2H), 1.11 (s, 6H), 0.88 (t, J = 7.9 Hz, 10H), 0.53 (p, J = 7.8 Hz, 6H). ^13^C NMR (126 MHz, DMSO-d6) δ 201.90 , 170.59 , 169.75 , 165.59 , 160.10 , 139.53 , 136.40 , 134.21 , 133.89 , 131.99 , 130.02 , 129.16 , 129.06 , 128.90 , 128.45 , 127.32 , 114.11 , 83.63 , 83.06 , 80.35 , 77.11 , 75.78 , 75.05 , 74.73 , 72.47 , 71.58 , 58.27 , 55.66 , 43.52 , 37.20 , 35.61 , 26.85 , 22.20 , 21.50 , 21.04 , 14.52 , 10.25 , 7.02 , 5.22 .

*Synthesis of compound PTX-2.* Compound 2d (500 mg, 0.5 mmol) was dissolved in 5 mL MeOH, followed by the addition of p-toluenesulfonic acid (PTS, 155 mg, 0.9 mmol). The reaction mixture was stirred at 50 °C for 5 h. After completion, the reaction was quenched by dilution with EtOAc, and the organic layer was sequentially washed with saturated NaHCO_3_ and brine. The combined organic layers were dried over anhydrous Na_2_SO_4_, and the solvent was removed under reduced pressure by rotary evaporation. The crude product was purified by column chromatography (EtOAc:PE = 2:1) to obtain white solid PTX-2 (240 mg), yield 50%.^1^H NMR (500 MHz, DMSO-d6) δ 9.08 (d, J = 8.5 Hz, 1H), 7.98 – 7.95 (m, 2H), 7.91 – 7.88 (m, 2H), 7.75 – 7.71 (m, 1H), 7.66 – 7.59 (m, 4H), 7.39 (d, J = 4.6 Hz, 5H), 7.21 (dd, J = 6.7, 3.1 Hz, 1H), 6.28 (d, J = 14.1 Hz, 2H), 5.88 (t, J = 8.8 Hz, 1H), 5.41 (d, J = 7.3 Hz, 1H), 5.37 (t, J = 8.3 Hz, 1H), 4.96 (d, J = 6.9 Hz, 1H), 4.94 – 4.90 (m, 1H), 4.72 (s, 1H), 4.58 (t, J = 7.6 Hz, 1H), 4.40 (s, 1H), 4.10 (dt, J = 11.1, 6.8 Hz, 1H), 4.01 (q, J = 8.3 Hz, 2H), 3.61 (d, J = 7.2 Hz, 1H), 2.38 – 2.28 (m, 1H), 2.22 (s, 3H), 2.12 (s, 3H), 1.86 (dd, J = 15.6, 9.3 Hz, 1H), 1.80 – 1.77 (m, 3H), 1.72 – 1.61 (m, 2H), 1.50 (s, 3H), 1.30 (s, 1H), 1.27 – 1.22 (m, 4H), 1.02 (d, J = 6.5 Hz, 6H). ^13^C NMR (126 MHz, DMSO-d6) δ 202.86 , 173.15 , 170.32 , 169.25 , 165.93 , 165.66 , 139.71 , 139.52 , 134.97 , 133.92 , 132.10 , 130.43 , 130.02 , 129.16 , 129.01 , 128.78 , 128.19 , 127.96 , 114.01 , 84.07 , 83.35 , 80.73 , 77.22 , 75.84 , 75.25 , 74.06 , 70.95 , 70.05 , 57.90 , 56.97 , 55.62 , 46.58 , 43.44 , 37.02 , 35.15 , 26.79 , 23.00 , 21.78 , 21.16 , 14.31 , 10.26 .

Synthesis of PTX-3 probe

*Synthesis of compound 3a.* Compound 2d (500 mg, 0.5 mmol) was dissolved in anhydrous THF (10 mL), followed by the dropwise addition of a 1.0 M TBAF/THF solution (94 μL, 0.4 mmol) at room temperature. The reaction mixture was stirred for 1 h. After completion, water and dichloromethane (DCM) were added for extraction. The combined organic layers were dried over anhydrous Na_2_SO_4_ and concentrated under reduced pressure by rotary evaporation. The crude product was purified by column chromatography (EtOAc:PE = 1:4) to afford white solid compound 3a (335 mg), yield 67%.^1^H NMR (500 MHz, DMSO-d6) δ 8.04 – 7.98 (m, 2H), 7.71 – 7.63 (m, 1H), 7.56 (t, J = 7.6 Hz, 2H), 7.44 – 7.37 (m, 6H), 7.28 (d, J = 8.0 Hz, 3H), 7.00 – 6.95 (m, 2H), 6.41 (s, 1H), 6.16 (t, J = 8.9 Hz, 1H), 5.52 (d, J = 7.1 Hz, 1H), 5.14 (s, 1H), 5.01 (d, J = 6.8 Hz, 1H), 4.95 (s, 1H), 4.92 – 4.88 (m, 1H), 4.34 (s, 1H), 4.18 (dt, J = 11.0, 6.9 Hz, 1H), 4.02 (d, J = 9.7 Hz, 2H), 3.79 (s, 3H), 3.77 (d, J = 7.2 Hz, 1H), 2.38 – 2.30 (m, 2H), 2.25 (dd, J = 15.2, 8.9 Hz, 1H), 2.16 (s, 3H), 2.13 (s, 3H), 2.04 – 2.00 (m, 3H), 1.70 – 1.60 (m, 2H), 1.54 (s, 3H), 1.12 (s, 3H), 1.11 (s, 3H). ^13^C NMR (126 MHz, DMSO-d6) δ 202.89 , 170.40 , 169.90 , 169.27 , 165.59 , 139.55 , 136.37 , 134.13 , 133.85 , 132.04 , 130.02 , 129.64 , 129.16 , 129.08 , 128.92 , 128.46 , 127.35 , 114.14 , 84.11 , 83.07 , 80.78 , 77.15 , 75.83 , 75.32 , 74.87 , 71.66 , 70.95 , 58.04 , 55.66 , 47.98 , 46.76 , 43.53 , 37.08 , 35.69 , 33.82 , 26.88 , 25.80 , 24.93 , 22.25 , 21.63 , 21.19 , 14.73 , 10.23 .

*Synthesis of compound 3b.* In a 100 mL round-bottom flask under an inert nitrogen atmosphere, compound 3a (200 mg, 0.2 mmol), 4-benzoylbenzoic acid (54.5 mg, 0.3 mmol), DCC (49.7 mg, 0.3 mmol), and DMAP (73.6 mg, 0.6 mmol) were sequentially added. The mixture was dissolved in dichloromethane (DCM, 10 mL) and stirred at room temperature for 6 h. After completion, the reaction mixture was diluted with DCM and sequentially washed with dilute hydrochloric acid, saturated NaHCO₃, and brine. The combined organic layers were dried over anhydrous Na_2_SO_4_ and concentrated under reduced pressure by rotary evaporation. The crude product was purified by column chromatography (EtOAc:PE = 1:2) to afford white solid compound 3b (140 mg), yield 60%.1H NMR (500 MHz, DMSO-d6) δ 8.06 – 8.02 (m, 2H), 7.99 – 7.96 (m, 2H), 7.86 – 7.81 (m, 2H), 7.79 – 7.76 (m, 2H), 7.75 – 7.67 (m, 2H), 7.62 – 7.57 (m, 4H), 7.41 (t, J = 5.7 Hz, 6H), 7.29 (d, J = 7.8 Hz, 2H), 6.99 (s, 1H), 6.32 (s, 1H), 6.20 (t, J = 8.9 Hz, 1H), 5.73 (dd, J = 10.4, 7.4 Hz, 1H), 5.60 (t, J = 7.8 Hz, 2H), 5.18 (s, 1H), 5.09 (s, 1H), 5.02 (d, J = 9.3 Hz, 1H), 4.34 (s, 1H), 4.12 (s, 2H), 3.97 (d, J = 6.9 Hz, 1H), 3.80 (s, 3H), 2.68 – 2.58 (m, 1H), 2.28 (dd, J = 15.2, 8.8 Hz, 1H), 2.18 (s, 3H), 2.04 – 2.01 (m, 3H), 1.99 (s, 3H), 1.85 (s, 3H), 1.73 (dd, J = 12.7, 4.2 Hz, 1H), 1.63 – 1.58 (m, 1H), 1.53 – 1.47 (m, 1H), 1.16 (s, 3H), 1.09 (s, 3H).^13^C NMR (126 MHz, DMSO-d6) δ 202.58 , 195.84 , 170.81 , 169.87 , 164.42 , 160.13 , 141.30 , 140.32 , 139.51 , 136.85 , 136.42 , 133.68 , 133.33 , 132.04 , 130.25 , 130.07 , 129.94 , 129.80 , 129.65 , 129.19 , 129.09 , 128.94 , 128.48 , 127.33 , 114.18 , 83.09 , 80.25 , 77.18 , 74.91 , 74.51 , 72.79 , 56.12 , 55.67 , 47.96 , 43.53 , 33.82 , 26.75 , 25.80 , 24.93 , 22.20 , 21.62 , 20.79 , 14.73 , 11.22 .

*Synthesis of compound PTX-3.* Compound 3b (500 mg, 0.4 mmol) was dissolved in methanol 5 mL MeOH, followed by the addition of p-toluenesulfonic acid (PTS, 107.3 mg, 0.6 mmol). The reaction mixture was stirred at 50 °C for 5 h. After completion, the reaction was quenched by dilution with ethyl acetate (EtOAc) and extracted with saturated NaHCO₃. The organic layer was washed with brine, dried over anhydrous Na_2_SO_4_, and concentrated under reduced pressure. The crude product was purified by column chromatography (EtOAc:PE = 1:1) to afford white solid PTX-3 (240 mg), yield 50%.^1^H NMR (500 MHz, DMSO-d6) δ 9.09 (d, J = 8.5 Hz, 1H), 8.01 (dt, J = 7.1, 1.4 Hz, 2H), 7.98 – 7.95 (m, 2H), 7.94 – 7.91 (m, 2H), 7.84 – 7.80 (m, 2H), 7.78 – 7.70 (m, 5H), 7.68 – 7.64 (m, 2H), 7.64 – 7.57 (m, 5H), 7.44 – 7.42 (m, 4H), 7.25 (ddd, J = 8.5, 5.1, 3.4 Hz, 1H), 6.27 (d, J = 7.4 Hz, 1H), 6.21 (s, 1H), 5.95 – 5.90 (m, 1H), 5.68 (dd, J = 10.4, 7.3 Hz, 1H), 5.51 (d, J = 7.1 Hz, 1H), 5.42 (t, J = 8.2 Hz, 1H), 5.07 – 5.03 (m, 1H), 4.87 (s, 1H), 4.63 (t, J = 7.7 Hz, 1H), 4.40 (s, 1H), 4.11 (q, J = 8.5 Hz, 2H), 3.83 (d, J = 7.0 Hz, 1H), 2.66 – 2.58 (m, 1H), 2.29 (s, 3H), 1.95 (s, 3H), 1.93 – 1.89 (m, 1H), 1.85 – 1.80 (m, 7H), 1.78 – 1.73 (m, 1H), 1.23 (s, 2H), 1.06 (s, 3H), 1.02 (d, J = 5.6 Hz, 4H). ^13^C NMR (126 MHz, DMSO-d6) δ 173.14 , 168.72 , 164.39 , 140.50 , 139.55 , 136.85 , 134.98 , 133.68 , 133.34 , 133.14 , 132.12 , 130.24 , 130.07 , 129.93 , 129.78 , 129.19 , 128.81 , 128.19 , 127.96 , 125.04 , 83.35 , 83.24 , 80.20 , 77.23 , 75.74 , 74.86 , 74.59 , 74.05 , 72.75 , 69.92 , 56.95 , 55.95 , 46.43 , 43.43 , 35.11 , 29.47 , 26.67 , 22.95 , 21.73 , 20.77 , 14.35 , 11.26 .

Synthesis of PTX-4 probe

*Synthesis of compound 4a.* 3-Amino-4-iodobenzoic acid methyl ester (500 mg, 1.8 mmol), copper(I) iodide (17.2 mg, 0.1 mmol), and tetrakis(triphenylphosphine)palladium (104.2 mg, 0.1 mmol) were sequentially added to a round-bottom flask and dissolved in anhydrous tetrahydrofuran15 mL. Under an inert nitrogen atmosphere, trimethylsilylacetylene (472 μL, 3.6 mmol) was quickly added, followed by the slow addition of triethylamine (526 μL, 3.6 mmol). The reaction mixture was stirred at room temperature overnight. The solvent was removed under reduced pressure. The residue was dissolved in EtOAc, washed sequentially with water and saturated brine, dried over anhydrous Na₂SO₄, and concentrated under reduced pressure. The crude product was purified by column chromatography (EtOAc:PE = 1:5) to afford compound 4a (332 mg), yield 67%.^1^H NMR (500 MHz, Chloroform-d) δ 7.37 (d, J = 1.4 Hz, 1H), 7.33 (s, 1H), 7.32 (d, J = 1.6 Hz, 1H), 3.88 (s, 3H), 0.28 (s, 9H). ^13^C NMR (126 MHz, Chloroform-d) δ 166.82 , 148.11 , 132.18 , 130.96 , 118.52 , 114.92 , 111.97 , 100.92 , 77.68 – 75.97 (m), 52.14 , 0.00 .

*Synthesis of compound 4b.* Compound 4b (30 mg, 0.12 mmol) and sodium methoxide (26.2 mg, 0.48 mmol) were dissolved in 1 mL methanol. The reaction mixture was stirred at room temperature for 1 h. After completion, the solvent was removed under reduced pressure by rotary evaporation. The residue was dissolved in EtOAc, washed sequentially with water (twice), dried over anhydrous Na_2_SO_4_, and concentrated under reduced pressure. The crude product was purified by column chromatography (EtOAc:PE = 1:5) to afford compound 4b (18 mg), yield 61%.^1^H NMR (400 MHz, Chloroform-d) δ 7.40 (d, J = 1.5 Hz, 1H), 7.38 (s, 1H), 7.35 (d, J = 1.5 Hz, 1H), 3.91 (s, 3H), 3.53 (s, 1H). ^13^C NMR (101 MHz, Chloroform-d) δ 166.78 , 148.40 , 132.59 , 131.33 , 118.55 , 115.08 , 84.86 , 79.91 , 77.91 – 76.34 (m), 52.24 .

*Synthesis of compound 4c.* Compound 4b (200 mg, 1.1 mmol) was dissolved in a mixture of acetonitrile (5 mL) and 10% aqueous H₂SO₄ solution (8 mL). A solution of sodium nitrite (118 mg, 1.6 mmol) in water (2 mL) was added dropwise at 0 °C, and the reaction mixture was stirred at 0 °C for 0.5 h. Subsequently, a solution of sodium azide (111 mg, 1.6 mmol) in water (2 mL) was added dropwise at 0 °C, and the reaction mixture was allowed to warm to room temperature and stirred for an additional 0.5 h. After completion, the reaction was quenched by dilution with water, and the product was extracted with EtOAc. The organic layer was washed with saturated brine, dried over anhydrous Na_2_SO_4_, and concentrated under reduced pressure. The crude product was purified by column chromatography (EtOAc:PE = 1:6) to afford compound 4c (185 mg), yield 91%.^1^H NMR (500 MHz, DMSO-d6) δ 7.72 (d, J = 1.1 Hz, 1H), 7.71 (dd, J = 4.0, 1.7 Hz, 1H), 7.65 – 7.62 (m, 1H), 4.75 (s, 1H), 3.89 (s, 3H). ^13^C NMR (126 MHz, DMSO-d6) δ 165.40 , 142.10 , 134.66 , 131.41 , 125.73 (d, J = 3.3 Hz), 119.93 (d, J = 7.0 Hz), 89.90 , 79.05 , 61.86 , 53.08 , 40.00 (dp, J = 42.2, 21.0 Hz), 14.54 .

Synthesis of compound 4d. Compound 4c (100 mg, 0.5 mmol) was dissolved in MeOH (10 mL), followed by the addition of LiOH·H₂O (41.7 mg, 0.9 mmol). The reaction mixture was stirred at room temperature for 1 h. After completion, the solvent was removed under reduced pressure. The residue was diluted with water, washed with EtOAc, acidified with dilute hydrochloric acid, and then extracted sequentially with EtOAc. The combined organic layers were dried over anhydrous Na_2_SO_4_ and concentrated under reduced pressure to afford crude compound 4d for the next step.

*Synthesis of compound 4e.* (2R,3S)-3-Amino-2-hydroxy-3-phenylpropanoic acid methyl ester (500 mg, 2.5 mmol), compound 4d (575 mg, 3.0 mmol), EDCI (736.5 mg, 3.8 mmol), and HOBT (519 mg, 3.8 mmol) were sequentially added to a 100 mL round-bottom flask and dissolved in anhydrous DMF (30 mL). DIEA (1.3 mL, 7.6 mmol) was slowly added dropwise to the reaction mixture, which was then stirred at room temperature for 5 h. After completion, the reaction was quenched by extraction with EtOAc, followed by sequential washing with dilute hydrochloric acid and saturated NaHCO₃. The combined organic layers were washed with saturated brine, dried over anhydrous Na_2_SO_4_, and filtered to remove the drying agent. The solvent was removed under reduced pressure, and the crude product was purified by column chromatography (EtOAc:PE = 1:2) to afford compound 4e (450 mg), yield 80%.^1^H NMR (400 MHz, DMSO-d6) δ 9.01 (d, J = 8.8 Hz, 1H), 7.72 (d, J = 1.4 Hz, 1H), 7.67 – 7.62 (m, 2H), 7.43 – 7.39 (m, 2H), 7.36 – 7.31 (m, 2H), 7.29 – 7.24 (m, 1H), 5.97 (d, J = 6.9 Hz, 1H), 5.41 (dd, J = 8.8, 5.7 Hz, 1H), 4.67 (s, 1H), 4.50 (t, J = 6.1 Hz, 1H), 3.52 (s, 3H). ^13^C NMR (126 MHz, DMSO-d6) δ 172.80 , 165.18 , 141.66 , 136.22 ,

134.24 , 128.62 , 127.80 , 118.79 , 88.89 , 79.30 , 73.99 , 56.75 , 55.38 , 52.08 , 39.98 (dp, J = 41.9, 21.0 Hz).

*Synthesis of compound 4f.* In a round-bottom flask, compound 4e (300 mg, 0.8 mmol) and pyridinium p-toluenesulfonate (PPTS) (20.7 mg, 0.08 mmol) were sequentially added and dissolved in anhydrous toluene (15 mL). Subsequently, under an inert nitrogen atmosphere, 4-methoxybenzaldehyde dimethyl acetal (210 μL, 1.2 mmol) was slowly added dropwise. The reaction mixture was refluxed for 1 hour. After completion, the mixture was extracted with EtOAc, and the combined organic phase was washed with saturated NaHCO₃ and saturated brine, followed by drying over anhydrous Na_2_SO_4_. The crude product was purified by column chromatography (EtOAc:PE = 1:5) to afford compound 4f (168 mg) yield 56%.^1^H NMR (400 MHz, DMSO-d6 ) δ 7.46 (d, J = 7.9 Hz, 2H), 7.36 (s, 6H), 7.09 (d, J = 7.8 Hz, 1H), 6.92 (s, 3H), 6.57 (s, 1H), 5.34 (s, 1H), 5.01 (s, 1H), 4.59 (s, 1H), 3.77 (s, 6H). ^13^C NMR (126 MHz, DMSO-d6) δ 170.18 , 160.14 , 152.84 , 141.48 , 134.37 , 131.58 – 127.21 (m), 123.46 , 117.74 , 114.06 , 88.40 , 79.11 , 55.63 , 53.02 , 40.00 (dp, J = 41.9, 20.9 Hz).

*Synthesis of compound 4g.* Compound 4f (100 mg, 0.2 mmol) was dissolved in MeOH (10 mL), and LiOH·H₂O (10.4 mg, 0.2 mmol) was slowly added. The reaction mixture was stirred at room temperature for 1 hour. After completion, MeOH was evaporated under reduced pressure. The mixture was diluted with water, extracted with EtOAc, and acidified with dilute hydrochloric acid. The organic phase was dried over anhydrous Na_2_SO_4_ and concentrated under reduced pressure to afford crude compound 4g, which was used directly in the next step without further purification.

*Synthesis of compound 4h.* Baccatin III-1 (600 mg, 0.9 mmol), modified side chain 4g (802 mg, 1.8 mmol), DCC (265 mg, 1.3 mmol), and DMAP (52 mg, 0.4 mmol) was sequentially added to a 50 mL round-bottom flask. Under an inert nitrogen atmosphere, the solids were dissolved in anhydrous 6 mL toluene. The reaction mixture was stirred at room temperature for 5 hours. After completion, the mixture was extracted with EtOAc. The organic phase was washed successively with saturated NaHCO₃and saturated brine, then dried over anhydrous Na_2_SO_4._ The solvent was removed under reduced pressure to obtain the crude product, which was purified by column chromatography (EtOAc:PE = 1:3) to afford white solid 4h (468 mg)，yield 76%.^1^H NMR (500 MHz, DMSO-d6) δ 8.00 (d, J = 7.5 Hz, 2H), 7.68 (t, J = 7.5 Hz, 1H), 7.56 (t, J = 7.7 Hz, 2H), 7.44 (d, J = 31.6 Hz, 7H), 7.06 (d, J = 89.0 Hz, 4H), 6.37 (s, 1H), 6.15 (t, J = 9.1 Hz, 1H), 5.51 (d, J = 7.1 Hz, 1H), 5.10 (d, J = 13.6 Hz, 1H), 4.96 (s, 1H), 4.91 (d, J = 9.5 Hz, 1H), 4.59 (s, 1H), 4.43 (dd, J = 10.5, 6.8 Hz, 1H), 4.03 (s, 2H), 3.80 (s, 3H), 3.75 (d, J = 7.1 Hz, 1H), 2.49 – 2.44 (m, 1H), 2.36 (d, J = 12.9 Hz, 1H), 2.22 (dd, J = 15.6, 8.5 Hz, 1H), 2.14 (s, 3H), 2.09 (s, 2H), 2.06 (s, 3H), 1.74 – 1.64 (m, 2H), 1.56 (s, 3H), 1.29 – 1.21 (m, 3H), 1.11 (s, 4H), 0.88 (t, J = 7.9 Hz, 9H), 0.53 (hept, J = 7.5 Hz, 6H).^13^C NMR (126 MHz, DMSO-d6) δ 201.91 , 170.60 , 165.61 , 140.48 (d, J = 235.3 Hz), 129.80 (d, J = 60.2 Hz), 129.18 , 127.32 , 114.20 , 83.64 , 80.35 , 77.14 , 72.48 , 71.69 , 58.27 , 55.53 (d, J = 40.4 Hz), 46.55 , 43.51 , 39.99 (dp, J = 41.8, 20.9 Hz), 37.21 , 33.82 , 26.04 (d, J = 198.4 Hz), 22.71 – 19.87 (m), 14.50 (d, J = 19.1 Hz), 10.24 , 7.01 , 5.24 .

Synthesis of compound PTX-4. Compound 4h (200 mg, 0.17 mmol) was dissolved in 4 mL methanol, followed by the addition of p-toluenesulfonic acid (PTS) (59.8 mg, 0.34 mmol). The reaction mixture was stirred at 50 °C for 5 hours. After completion, the mixture was extracted with EtOAc. The organic phase was washed successively with saturated NaHCO₃ and saturated brine, then dried over anhydrous Na_2_SO_4_. The solvent was removed under reduced pressure to obtain the crude product, which was purified by column chromatography (EtOAc:PE = 2:1) to afford white solid PTX-4 (102 mg), yield 48%.^1^H NMR (400 MHz, DMSO-d6) δ 9.16 (d, J = 8.6 Hz, 1H), 8.00 – 7.95 (m, 2H), 7.73 (q, J = 2.9 Hz, 2H), 7.71 – 7.68 (m, 1H), 7.66 – 7.61 (m, 3H), 7.41 (d, J = 4.4 Hz, 4H), 7.24 (h, J = 4.2 Hz, 1H), 6.30 (s, 1H), 6.27 (d, J = 7.3 Hz, 1H), 5.90 (t, J = 9.1 Hz, 1H), 5.42 (t, J = 8.2 Hz, 2H), 4.97 – 4.89 (m, 2H), 4.73 (s, 1H), 4.68 (s, 1H), 4.60 (t, J = 7.5 Hz, 1H), 4.11 (dt, J = 10.9, 6.9 Hz, 1H), 4.02 (q, J = 8.2 Hz, 2H), 3.62 (d, J = 7.2 Hz, 1H), 2.37 – 2.26 (m, 1H), 2.23 (s, 3H), 2.12 (s, 3H), 2.03 – 1.97 (m, 1H), 1.91 (dd, J = 15.2, 9.4 Hz, 1H), 1.81 – 1.77 (m, 3H), 1.76 – 1.70 (m, 1H), 1.68 – 1.60 (m, 2H), 1.51 (s, 3H), 1.25 (d, J = 6.2 Hz, 2H), 1.03 (d, J = 3.9 Hz, 6H). ^13^C NMR (101 MHz, DMSO-d6) δ 202.87 , 173.11 , 170.33 , 169.26 , 165.64 , 165.23 , 141.74 , 139.67 , 139.33 , 134.27 , 133.82 , 130.42 , 130.05 , 129.19 , 128.83 , 127.95 , 124.33 , 118.79 , 116.56 , 88.98 , 84.06 , 80.71 , 79.31 , 77.20 , 75.07 (d, J = 30.5 Hz), 73.94 , 70.94 , 57.88 , 56.97 , 46.56 , 43.44 , 40.74 – 39.26 (m), 26.78 , 23.00 , 21.48 (d, J = 61.8 Hz), 14.31 , 10.26 .

# Supplementary Figures

Figure S1. Functional validation of the multidrug resistance (MDR) phenotypes through intracellular substrate accumulation assays.

Figure S2. (A) In-gel fluorescence analysis of live MCF-7 and MCF-7/PTX cells labeled with PTX-1 probe at different concentrations. (B) In-gel fluorescence analysis of live MCF-7 and MCF-7/PTX cells labeled with PTX-1 probe for different incubation time. (C) In-gel fluorescence analysis of live MCF-7 and MCF-7/PTX cells labeled with PTX-2 probe at different concentrations. (D) In-gel fluorescence analysis of live MCF-7 and MCF-7/PTX cells labeled with PTX-2 probe for different incubation time. FL, fluorescence imaging; CBB, Coomassie Brilliant Blue staining.

Figure S3. (A) In-gel fluorescence analysis of live MCF-7 and MCF-7/PTX cells labeled with PTX-3 probe at different concentrations. (B) In-gel fluorescence analysis of live MCF-7 and MCF-7/PTX cells labeled with PTX-3 probe for different incubation time. (C) Competitive inhibition of PTX-2 and PTX-3 probes by paclitaxel in live MCF-7 and MCF-7/PTX cells. FL, fluorescence imaging; CBB, Coomassie Brilliant Blue staining.

Figure S4. Venn diagram and scatter plot analysis of PTX-4 probe-enriched proteins in MCF-7 and MCF-7/PTX cells.

Figure S5. (A) KEGG analysis of PTX-4 probe-enriched drug resistance-related proteins. (B) GO analysis of PTX-4 probe-enriched drug resistance-related proteins.

Figure S6. Biochemical validation of the PTX and AKT2 interaction via ADP-Glo kinase assay.

Figure S7. In vitro tubulin polymerization assay characterizing the influence of AKT2 on paclitaxel activity.

Figure S8. Impact of upstream and parallel pathway inhibition on PTX sensitivity in MCF-7 cells. Cell viability curves of parental WT/MCF-7 and resistant MCF-7/PTX cells treated with PTX alone or in combination with the PI3K inhibitor Alpelisib or the MAPK inhibitor Ralimetinib. The data demonstrate that pharmacological inhibition of the upstream PI3K pathway or the parallel MAPK pathway significantly resensitizes the resistant cells to PTX treatment. These findings further substantiate that the AKT2 centered signaling node is integrated into a broader network of resistance-associated pathways. Data are presented as mean ± SD, *n* = 3.

Figure S9. Uncropped blot for Figure 4d.

Figure S10. Uncropped blot for Figure 4e.

Figure S11. Uncropped blot for Figure 4f.

Figure S12. Uncropped blot for Figure 6a. The full, uncropped membranes for AKT2 (left) and GAPDH (right) internal control are presented, with red rectangles indicating the specific regions cropped for the main text figure. Note that the two membranes were run in the same gel electrophoresis and derived from the same batch of biological samples, but their respective lane positions do not align symmetrically due to the pre-loaded protein molecular weight markers. Specifically, for the AKT2 membrane (left), the six lanes between the two markers from left to right represent siNC, siNC, siAKT2, siNC, siAKT2, and siNC, respectively, while the lanes to the right of the second marker represent technical replicates. For the GAPDH membrane (right), the five sequential lanes between the markers directly correspond to lanes 2 to 6 of the AKT2 membrane (siNC, siAKT2, siNC, siAKT2, and siNC) from the identical experimental run.

# Table S1 PTX target proteins identified by PTX-4 probe in MCF-7 cells

# Table S2 PTX target proteins identified by PTX-4 probe in MCF-7/PTX cells

# NMR Spectrums

^1^H NMR of compound 1a

^13^C NMR of compound 1a

^1^H NMR of compound 1b

^1^H NMR of compound PTX-1

^13^C NMR of compound PTX-1

^1^H NMR of compound Baccatin III-1

^13^C NMR of compound Baccatin III-1

^1^H NMR of compound 2a

^13^C NMR of compound 2a

^1^H NMR of compound 2b

^13^C NMR of compound 2b

^1^H NMR of compound 2d

^13^C NMR of compound 2d

^1^H NMR of compound PTX-2

^13^C NMR of compound PTX-2

^1^H NMR of compound 3a

^13^C NMR of compound 3a

^1^H NMR of compound 3b

^13^C NMR of compound 3b

^1^H NMR of compound PTX-3

^13^C NMR of compound PTX-3

^1^H NMR of compound 4a

^13^C NMR of compound 4a

^1^H NMR of compound 4b

^13^C NMR of compound 4b

^1^H NMR of compound 4c

^13^C NMR of compound 4c

^1^H NMR of compound 4e

^13^C NMR of compound 4e

^1^H NMR of compound 4f

^13^C NMR of compound 4f

^1^H NMR of compound 4h

^13^C NMR of compound 4h

^1^H NMR of compound PTX-4

^13^C NMR of compound PTX-4
